# Aerobic anoxygenic phototrophs play important roles in nutrient cycling within cyanobacterial *Microcystis* bloom microbiomes

**DOI:** 10.1186/s40168-024-01801-4

**Published:** 2024-05-13

**Authors:** Haiyuan Cai, Christopher J. McLimans, Helong Jiang, Feng Chen, Lee R. Krumholz, K. David Hambright

**Affiliations:** 1 School of Biological Sciences, University of Oklahoma, Norman, USA; 2 Nanjing Institute of Geography and Limnology, Chinese Academy of Sciences, Nanjing, China; 3 Institute of Marine and Environmental Technology, University of Maryland Center for Environmental Science, Baltimore, USA

**Keywords:** AAP bacteria, Microbiome, Interactome, Metabolic complementarity, Symbiosis, Biochemical cycling

## Abstract

**Background:**

During the bloom season, the colonial cyanobacterium *Microcystis* forms complex aggregates which include a diverse microbiome within an exopolymer matrix. Early research postulated a simple mutualism existing with bacteria benefitting from the rich source of fixed carbon and *Microcystis* receiving recycled nutrients. Researchers have since hypothesized that *Microcystis* aggregates represent a community of synergistic and interacting species, an interactome, each with unique metabolic capabilities that are critical to the growth, maintenance, and demise of *Microcystis* blooms. Research has also shown that aggregate-associated bacteria are taxonomically different from free-living bacteria in the surrounding water. Moreover, research has identified little overlap in functional potential between *Microcystis* and members of its microbiome, further supporting the interactome concept. However, we still lack verification of general interaction and know little about the taxa and metabolic pathways supporting nutrient and metabolite cycling within *Microcystis* aggregates.

**Results:**

During a 7-month study of bacterial communities comparing free-living and aggregate-associated bacteria in Lake Taihu, China, we found that aerobic anoxygenic phototrophic (AAP) bacteria were significantly more abundant within *Microcystis* aggregates than in free-living samples, suggesting a possible functional role for AAP bacteria in overall aggregate community function. We then analyzed gene composition in 102 high-quality metagenome-assembled genomes (MAGs) of bloom-microbiome bacteria from 10 lakes spanning four continents, compared with 12 complete *Microcystis* genomes which revealed that microbiome bacteria and *Microcystis* possessed complementary biochemical pathways that could serve in C, N, S, and P cycling. Mapping published transcripts from *Microcystis* blooms onto a comprehensive AAP and non-AAP bacteria MAG database (226 MAGs) indicated that observed high levels of expression of genes involved in nutrient cycling pathways were in AAP bacteria.

**Conclusions:**

Our results provide strong corroboration of the hypothesized *Microcystis* interactome and the first evidence that AAP bacteria may play an important role in nutrient cycling within *Microcystis* aggregate microbiomes.

Video Abstract

**Supplementary Information:**

The online version contains supplementary material available at 10.1186/s40168-024-01801-4.

## Introduction

Harmful algal blooms caused by cyanobacteria in freshwater lakes are a global ecological problem [1, 2]. Eutrophication, rising CO_2_ levels, and global warming are likely to increase cyanobacterial bloom frequency, intensity, and duration in aquatic ecosystems across the globe [3, 4]. *Microcystis* spp. are arguably the most important bloom-forming cyanobacteria in freshwater systems, due to their global distribution, being reported on every continent except Antarctica [5], as well as their ability to produce toxins, which is known to have caused the shutdown of drinking water sources [6, 7]. They form large colonies within amorphous mucilaginous sheathes [8] that constitute a niche for an abundant and diverse heterotrophic bacterial community [9–11] which together with *Microcystis* colonies comprise *Microcystis*-heterotrophic bacteria aggregates, hereafter *Microcystis* aggregates.

*Microcystis* aggregates constitute a unique physiochemical environment that likely supports proliferation of specific groups of bacteria. In addition to the rich variety of dissolved and particulate organic matter (DOM and POM) provided by *Microcystis* and their extracellular polymeric substance (EPS) matrix [12], potentially available as energy sources, the large size of aggregates can provide protection from zooplankton grazers [13–15], as well as from viral and bacterial threats [16]. Dissolved oxygen (DO) concentrations and pH within blooms fluctuate diurnally. For example, Chen and colleagues [17, 18] measured DO fluctuations from 8.0 mg L^−1^ during the day to 0.5 mg L^−1^ at night and pH from 9.0 during the day to 7.3 at night. Moreover, *Microcystis* possesses gas vesicles [19] that provide buoyancy to the aggregates, allowing seasonal and diurnal migration to the water’s surface and thus access to sunlight [19, 20].

This unique niche inhabited by heterotrophs surrounding phototrophs such as, but not limited to, *Microcystis* has been termed the phycosphere [21, 22]. Some researchers have postulated that algal–bacterial mutualisms may enhance the growth conditions for both cyanobacteria and associated bacteria [22, 23]. Further studies have suggested that cyanobacteria and associated bacteria may constitute functional interactomes [11, 24] in which multiple microbial constituents contribute to complete metabolic pathways. Such relationships have been corroborated by demonstrating tighter network connections between *Microcystis* and heterotrophic bacteria within aggregates compared to those between *Microcystis* and free-living bacteria [25, 26].

Previous work focusing on the diversity and function of isolates from cyanobacterial aggregates identified multiple novel species of aerobic anoxygenic phototrophic (AAP) bacteria [27–30] which have been hypothesized to play various roles in biogeochemical cycling within *Microcystis* bloom aggregates [25, 31–33]. For example, nutrient bioassays have demonstrated that *Microcystis* blooms can become nitrogen (N) limited during summer months, with internal cycling necessary to sustain a bloom [34, 35]. Studies based on microarrays and meta-transcriptomics have revealed denitrification and nitrogen fixation activities among associated bacteria in *Microcystis* blooms [32, 36]. Other reported copy numbers of denitrification-related genes were strongly correlated with *Microcystis* biomass [37]. Phosphorus (P) is also needed for *Microcystis* growth, and cyanobacteria are often considered less effective than green algae in competing for P when its availability is limited [38], yet high concentrations of dissolved organic phosphorus (DOP) in laboratory culture experiments inhibited *Microcystis* growth [33]. Yuan et al. [31] postulated that phosphorus regeneration by associated bacteria within *Microcystis* aggregates is more important than P assimilated directly from outside the aggregate, suggesting the importance of associated bacteria in providing phosphorus for *Microcystis* growth. Sulfate (SO_4_^−2^) is the primary sulfur (S) source for *Microcystis* and can be directly reduced by *Microcystis* through assimilatory pathways to produce organic sulfur compounds [39]. Organic sulfur compounds, such as dimethyl sulfide and dimethyl trisulfide, are excreted by live *Microcystis* cells in addition to release upon cell death [40]. As these sulfur compounds are degraded, sulfide is released, acting as a potential toxin to cyanobacteria [41], with the possible inhibition of cyanobacterial growth [42, 43]. These above studies, and others [44, 45], provide the framework for understanding the critical role of the *Microcystis* microbiome in nutrient cycling within bloom aggregates. However, mechanisms, metabolic pathways, and predominant taxa involved in nutrient cycling within the *Microcystis* interactome are poorly understood. If indeed *Microcystis* and members of microbiome constitute an interactome, functionally cooperating in C, N, S, and P cycling dynamics, one would expect to find complementary components of the various metabolic pathways required for such cycling.

Here, we test the hypothesis that *Microcystis* and members of its microbiome possess complementary genes coding for metabolic pathways that support nutrient cycling within *Microcystis* aggregates. Using a 7-month metagenomic survey of free-living and aggregate-associated bacterial assemblages in Lake Taihu, China, we found that most bacteria enriched in *Microcystis* aggregates were AAP bacteria (9 of 13 genera), while AAP bacteria were present at lower relative concentrations in the surrounding water. Then, using a comparative genome analysis of 102 high-quality bloom-associated bacterial metagenome-assembled genomes (MAGs) from 10 lakes spanning four continents, we found that the biochemical pathways coded in AAP bacteria and *Microcystis* were potentially complementary for their roles in C, N, S, and P cycling within *Microcystis* bloom aggregates. Analysis of relative expression patterns of biochemical pathways from published metatranscriptomes revealed that biochemical pathways in AAP bacteria associated with C, N, S, and P cycling were among the most active processes during *Microcystis* blooms. Our analyses support hypothesized complementarity between *Microcystis* and members of its microbiome, particularly AAP bacteria, and provide ample targets for future research opportunities to better understand the *Microcystis* interactome.

## Methods

### Metagenome survey of *Microcystis* aggregate-associated bacteria in Lake Taihu

Frequent *Microcystis* blooms occur annually in Lake Taihu, China, especially in Meiliang and Zhushan Bays throughout late spring through autumn [46]. *Microcystis* bloom samples were collected monthly from the surface water at two sites in Meiliang Bay (site 1: 31°30′N, 120°11′E; site 2: 31°24′N, 120°10′E) and at two sites in Zushan Bay (site 3: 31°27′N, 120°01′E; site 4:31°23′N, 120°00′E) (Fig. S1) from April to October in 2018. Samples were retrieved by dipping a sterile beaker off the side of a boat from the surface down to a depth of about 10 cm. Samples (2.5 L) from both sites of each bay were combined for subsequent manipulations. Subsamples were transferred into three 500-mL beakers and kept at room temperature for 10 min to allow cyanobacterial aggregates to float to the surface in each beaker. To obtain aggregate-microbiome bacteria, about 100 of the largest aggregates (1 ~ 2-mm diameter) of each sample in the floating aggregate layer were individually picked with a sterilized inoculation needle and were subjected to three successive sterile lake water washes (0.5 min each wash) [28] to detach free-living bacteria and loosely attached bacteria. The washed aggregates were combined and frozen prior to DNA extraction. The floating aggregate layer was then discarded, and the remaining 300 mL of water was filtered through a sterile 10-μm nylon net filter (Millipore) to remove any remaining aggregates [10]. The filtrate was filtered again through 0.2-μm pore-size filters to obtain the free-living bacteria fraction. Biomass on the filters was stored at − 80 °C before DNA extraction.

Temperature, DO, and pH were determined in situ using a YSI 6600 multiparameter water quality sensor. Diel changes in DO and pH in surface waters were measured in situ at site 1 in Meiliang Bay over a 24-h period from 10 to 10 AM on 10–11 August and 10–11 October 2018, corresponding to bloom peak and decline periods.

Genomic DNA was extracted using two methods in parallel to reduce possible extraction bias [47]: the UltraClean Soil DNA Isolation Kit (MoBio Laboratories, Carlsbad, CA, USA), and a phenol–chloroform protocol [48]. The concentration and purity of DNA were determined using a NanoDrop ND-2000 UV–Vis spectrophotometer (NanoDrop, Wilmington, DE, USA). DNA samples obtained by both methods were pooled in equal concentrations before further PCR and sequence analyses.

The 16S rRNA genes were amplified using 515F and 907R primers [49]. Sequencing of the 16S rRNA genes was performed using the Illumina MiSeq platform at Meiji Biotechnology Company (Shanghai, China). Amplicon sequences (16S) were deposited in the NCBI Sequence Read Archive under accession numbers (BioProject ID PRJNA985885) (Table S1) and processed through the QIIME2 pipeline and its associated modules [50]. Briefly, an amplicon sequence variant (ASV) table was inferred using the DADA2 pipeline of QIIME2. Taxonomic annotation of ASVs was done using the SILVA database v138 [51]. The ASV table was filtered to remove mitochondria, chloroplasts, Eukarya, and cyanobacteria. Alpha-diversity indices for PD faith metrics and beta-diversity indices for weighted UniFrac distances were calculated with QIIME2 plug-ins using the filtered ASVs. Phylogenetic structure dissimilarities were compared for aggregate-associated and free-living assemblages using the weighted UniFrac distance and displayed in principal coordinates analysis (PCoA) plots. Differences between the two bacterial assemblages were tested using the Adonis test. ASV counts were aggregated to genus, and Welch’s *t*-test implemented in STAMP [52], to identify the bacterial genera (*p*-value < 0.05 corrected by Benjamini–Hochberg FDR) for which relative abundances differed significantly between the aggregate-associated and the free-living communities.

### Generality of AAP bacteria in *Microcystis* microbiomes

To examine whether AAP bacteria were generally important constituents of *Microcystis* blooms beyond Lake Taihu, we analyzed published *Microcystis*-bloom metagenomes from 10 global lakes [11, 53]. The lakes spanned 90° latitude from Lake Aasee, Germany (52.0°N), to Lake Rotoehu, New Zealand (38.0°S), and 274° longitude from Castlerock Pond, USA (97.5°W), to Lake Rotoehu (176.5°E). General limnological information was included in previous studies [11, 53]. All metagenomic sequences are available in GenBank (BioProject accession number PRJNA575023) [11, 53] and were used here. Shotgun sequencing of the 10 global lakes and analysis, including quality trimming, removal of cyanobacterial reads, assembly, and MAG binning, is described in Cook et al. [11]. The quality of these bacterial MAGs was measured using CheckM v1.1.3. MAGs were further refined by manual removal of contamination using VizBin v1.0 [54]. High- and middle-quality MAGs were selected with a threshold of < 5% contamination and > 80% completeness.

### Metabolic pathways inmicrobiome bacteria

A nonredundant Microbiome Genome Database was constructed using the 10 lake MAGs. It was constructed using “dereplicate” function of dRep v3.4.2 on the MAGs, based on > 30% aligned fraction and a genome-wide ANI threshold of 95% (− nc 0.3, − sa 0.95) [55], as described for the glacier [56] and human gut microbiome databases [57]. All MAGs were annotated using METABOLIC-G v4.0 [58] and DRAM v1.3 [59]. Based on the presence of photosynthetic gene clusters (PGCs) evidenced by the *bch*, *puf*, and *acsF* marker genes, a total of 49 MAGs with PGCs were categorized as AAP bacteria, and 53 lacking PGCs were categorized as non-AAP bacteria. MAGs were classified by GTDB-Tk v0.1.3 [60]. A total of 104 metabolic pathways were identified in these MAGs by METABOLIC-C v4.0 [58] to determine the presence and absence of pathways in AAP bacteria and non-AAP MAGs.

The pathways that exhibited significant differences between AAP and non-AAP MAGs were determined using a two-sample Kolmogorov–Smirnov test. This test compared the presence and absence of pathways in AAP and non-AAP MAGs, with a statistical significance threshold set at *p* < 0.05.

### Metabolic gene abundances in *Microcystis* and microbiome bacteria

To calculate the relative abundances of nutrient cycling genes within the aggregate community (i.e., microbiome + *Microcystis*), we added 12 complete *Microcystis* genomes to the Microbiome Genome Database, hereafter the Aggregate Genome Database. Nine of the *Microcystis* genomes were previously used in a pangenome analysis of *Microcystis* phylogeny [61], and three were recently released (*Microcystis aeruginosa* FACHB-905 (accession number: CP089094.1), *M. aeruginosa* NIBR18 (CP086723.1) [62], and *M. aeruginosa* NIES-88 (AP024565.1). Gene prediction of the aggregate genome database was performed using Prodigal v2.6.3 with the “ − p meta” option. Gene functions were annotated using “kegg_annotation” function of Diting v0.9 [63] by querying the translated protein sequences against the KOfam database (ftp://ftp.genome.jp/pub/db/kofam) using hmmsearch [64] with KOfam suggested threshold values [65].

Specific gene abundances in the metagenomes were obtained by mapping the concatenated reads of all 10 lake samples back to the predicted gene sequences of the aggregate genome database using BWA-MEM with default settings [66] to generate sequence alignment map (SAM) files. The SAM files were used as input for pileup.sh of BBMap v38.22 with default settings [67] to calculate the average coverage of each gene. The GPM (genes per million) values for predicated genes were calculated as a proxy for gene abundance using the “table_of_ko_abundance_among_samples” function of DiTing v0.9 [63] with the following formula:

$${GPM}_i=\frac{b_i}{\sum_jb_j}\;\cdot\;10^6=\frac{\frac{X_i}{L_i}}{\sum_j\frac{X_j}{L_j}}\;\cdot\;10^6$$

where GPM_i_ is the relative abundance of gene i, b_i_ is the copy number of gene i, L_i_ is the length of gene i, X_i_ is the number of times that gene i is detected in a sample (i.e., the number of reads in alignment), and j is the number of genes in a sample. This relative measure of abundance was developed for quantifying gene transcripts, as TPM (transcripts per million) [68], but is also equally useful in metagenomics studies [69]. GPM enables comparisons of gene abundances across different samples by normalizing for variations in sequencing length and depth, ensuring that each sample has the same number of total counts [68].

The GPMs were used to quantify relative abundances of specific biochemical pathways using formulae suggested by DiTing v0.9 [63]. For example, assimilatory sulfate reduction converting sulfite to sulfide has two known possible pathways: (1) the *cysJI* (K00380 and K00381) (encoding sulfite reductase (NADPH) flavoprotein alpha and beta-component)-mediated pathway [70] and (2) the *sir* (K00392) (encoding sulfite reductase (ferredoxin))-mediated pathway [71]. Thus, the relative abundance of the assimilatory sulfate reduction pathway is estimated by the following formula:

$${\text{GPM}}_{\mathrm{assimilatory}\;\mathrm{sulfate}\;\mathrm{reduction}}=({\text{GPM}}_{\text{K}00380}+{\text{GPM}}_{\text{K}00381})/2\;+\;{\text{GPM}}_{\text{K}00392}$$

Due to the lack of relative abundance calculations for the P cycling pathways in DiTing v0.9 [63], functional genes related to P cycling were selected from within KEGG modules [72].

### AAP and non-AAP bacterial metatranscriptomes

To investigate hypothesized functional roles of microbiome bacteria in *Microcystis* aggregates, we leveraged publicly available (NCBI SRA database and the MG-RAST [73] server) (Table S2) *Microcystis* bloom metatranscriptomes from Lake Taihu and western Lake Erie and calculated the relative abundances of microbiome genes potentially involved in specific biochemical pathways. Metatranscriptomes of Lake Erie were collected at 7 sites in October 2013 (PRJNA262053), 14 sites in July and August 2014 (PRJNA354726), and a microcosm study in July 2019 (PRJNA823389). Metatranscriptomes of Lake Taihu were collected at one site in May 2015 (PRJNA359157) [32], one site from June to October 2015 (PRJNA664620), and one site from July to October 2016 (mgp103977) [74]. Because we lack specific microbiome genomes from the metatranscriptome studies, a general *Microcystis* microbiome genome set was created from 546 *Microcystis* bloom-associated microbiome genomes including MAGs and isolate genomes from western Lake Erie [75], Lake Taihu [44], Lake Champlain, and Pampulha reservoir [23]. Low-quality MAGs (completeness < 95%, contamination > 5%) were identified and removed using CheckM v1.1.3. The remaining MAGs were then dereplicated using dRep v3.4.2 with the same settings as for metagenome analysis described above to obtain a nonredundant microbiome genome set (Table S3) consisting of 122 high-quality microbiome MAGs, which were annotated by DRAM v1.3 [59]. All genomes with PGCs were considered AAP bacteria genomes. This genome set was combined with the microbiome genome database created with the 10 global lake MAGs described above to generate an expanded microbiome genome database. Gene prediction and annotation for the expanded microbiome genome database were conducted using the same methods as the metagenome analysis described above.

The bloom transcriptomes from Lake Erie and Lake Taihu were then mapped to the genes in the expanded microbiome genome database and categorized as being derived from either AAP or non-AAP bacteria. Relative expression (TPM) of KEGG orthologs (KOs) and biogeochemical pathways were estimated as described above for relative gene abundances. The relative abundances of pathways from AAP and non-AAP groups were represented in box plots constructed through *geom_boxplot* and *geom_jitter geom* in the package in R v3.3.6. Significance tests for comparisons of pathway expression between the AAP and non-AAP groups were performed using a nonparametric pairwise Wilcoxon test (*p*-value < 0.05 corrected by Benjamini–Hochberg FDR) within the stat_compare_means function in ggpubr v0.4.0.

As different metabolic processes are known to predominate during daytime and nighttime activities in *Microcystis* blooms [76], we leveraged publicly available (NCBI SRA database: SRP117911, SRP117914, SRP117915, SRP117922, SRP128942, SRP128945, and SRP128954) transcripts from a diel study of *Microcystis* blooms in western Lake Erie during late August 2014 (Table S4) [76]. These transcripts were mapped against the expanded microbiome genome database and 12 complete *Microcystis* genomes for metagenome analysis described above and categorized as being derived from either AAP, non-AAP bacteria, or *Microcystis* as described above.

## Results

### *Microcystis* microbiome bacteria in Lake Taihu

Chl *a* data indicated a rapid development of the cyanobacterial bloom in Lake Taihu, with peak abundance in August, followed by a decline in September and October (Table S5). Chl *a* concentrations were linearly correlated with DOC concentrations in bloom samples (Fig. S2). During the bloom peak, total nitrogen concentrations reached their lowest values (Table S5). Diel changes of DO and pH within the bloom (Fig. S3) during peak and decline phases likely influenced the expression of some biogeochemical pathways.

*Microcystis* 16S rRNA gene reads made up 65 ± 14% (mean ± standard deviation) of reads in the Lake Taihu aggregate-associated community during the 7-month study, while the free-living bacterial community contained only 2 ± 1% *Microcystis* reads. Non-cyanobacterial α-diversity was significantly lower (*p* < 0.001) in aggregates compared with free-living assemblages (Fig. S4A). *β*-diversity was also significantly different between aggregate and free-living assemblages (weighted UniFrac (*p* < 0.001) (Fig. S4B).

**Fig. 1:**
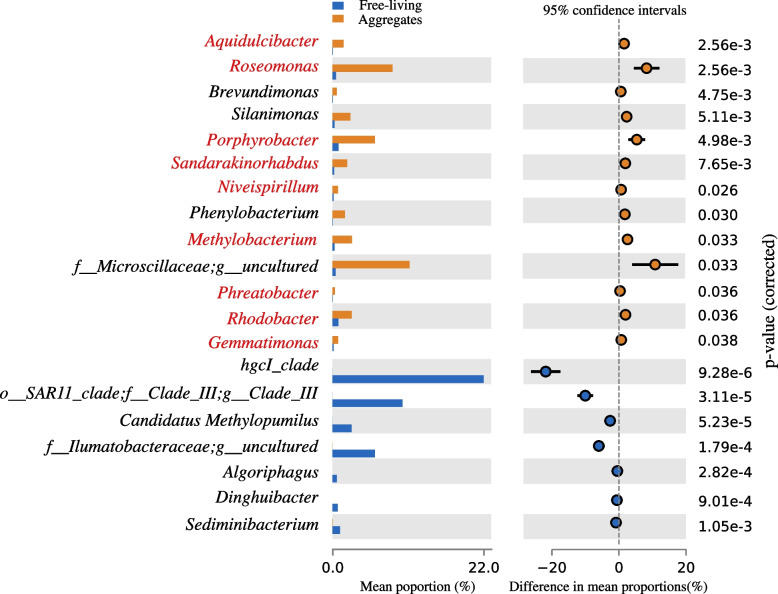
Genera that differed significantly between the aggregate and free-living communities. The left-hand panel shows the relative abundance (percent of total) of each genus in free-living (blue) and aggregate (orange) samples, while the right-hand panel shows the mean differences in proportion between the two communities. AAP bacterial genera are in red type. Welch’s *t*-test was implemented in STAMP [52], with *p*-values corrected with Benjamini–Hochberg FDR method [52]

Thirteen genera, including 9 AAP bacterial genera (*Aquidulcibacter*, *Roseomonas*, *Porphyrobacter*, *Sandarakinorhabdus*, *Niveispirillum*, *Methylobacterium*, *Phreatobacter*, *Rhodobacter*, and *Gemmatimonas*) and 3 non-AAP genera (*Brevundimonas*, *Silanimonas*, and *Phenylobacterium*) along with uncultured Microscillaceae were enriched in Lake Taihu aggregates relative to free-living communities (Fig. 1). Seven genera (all non-AAP bacteria) were enriched in the free-living community relative to aggregate communities. AAP bacteria abundances in Lake Taihu aggregates comprised 17 to 36% of the non-cyanobacteria aggregate microbial community but only 0.01 to 9.8% of the free-living bacterial community (Fig. S5).

### AAP bacteria in global *Microcystis* microbiomes

**Fig. 2:**
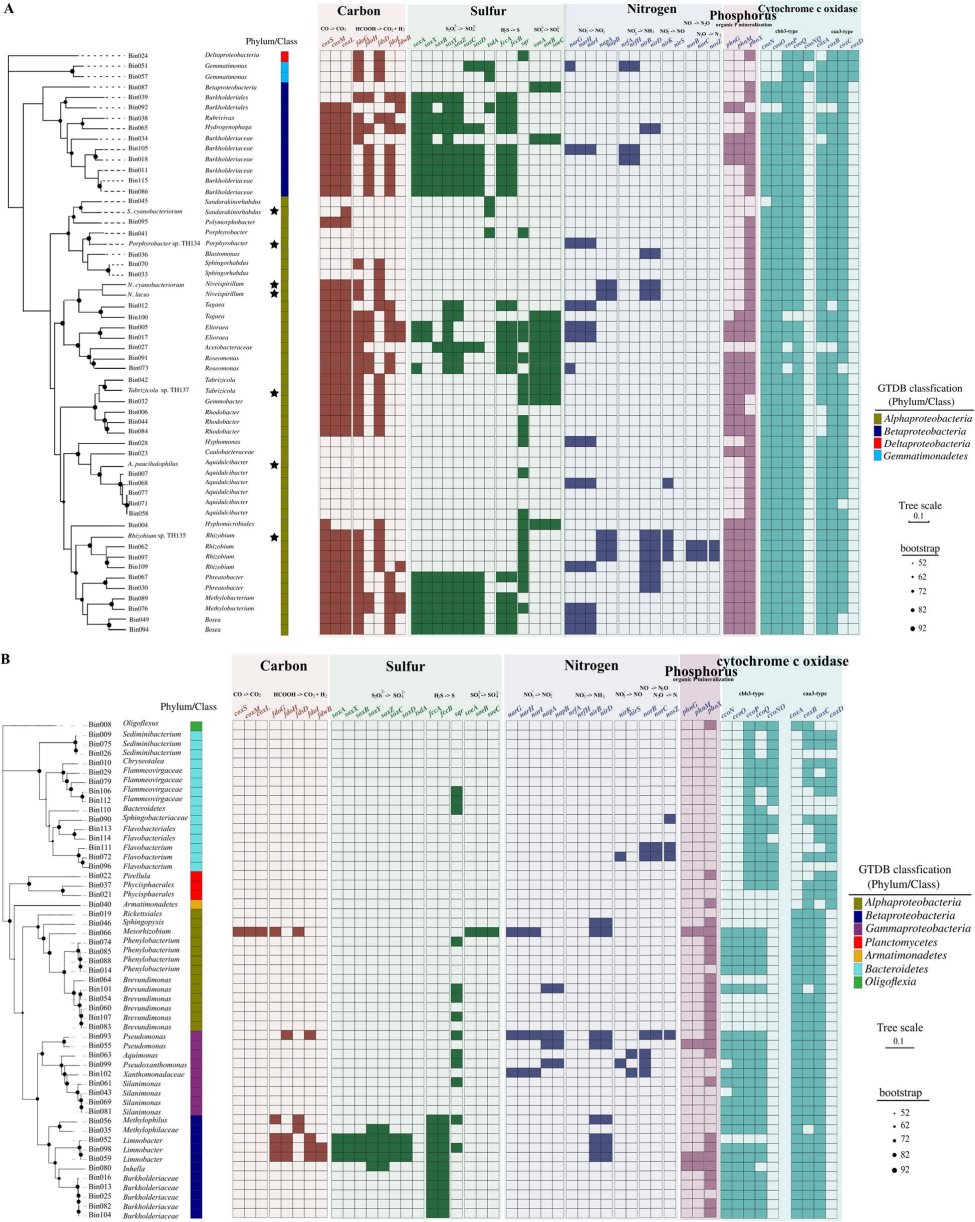
The presence (filled) and absence (blank) of genes associated with C, N, S, and P cycling pathways in AAP (**A**) and non-AAP (**B**) bacterial MAGs derived from bloom sample metagenomes. The phylogenetic trees were built using UBCG [77]. MAGs were classified by GTDB-Tk v0.1.3 [60]. Star indicates AAP bacterial isolates from *Microcystis* aggregates collected from Lake Taihu. Carbon cycling genes: *coxS*, *coxM*, and *coxL* (small, medium, and large subunit of aerobic carbon-monoxide dehydrogenase), *fdoG* (formate dehydrogenase major subunit), *fdoH* (formate dehydrogenase iron-sulfur subunit), *fdsD* (formate dehydrogenase delta subunit), *fdoI* (formate dehydrogenase gamma subunit), *fdwB* (formate dehydrogenase beta subunit). Sulfur cycling genes: *soxA* (sulfur-oxidizing protein SoxA), *soxX* (sulfur-oxidizing protein SoxX), *soxB* ( sulfur-oxidizing protein SoxB), *soxY* (sulfur-oxidizing protein SoxY), *soxZ* (sulfur-oxidizing protein SoxZ), *soxC* (S-disulfanyl-L-cysteine oxidoreductase SoxC), *soxD* (S-disulfanyl-L-cysteine oxidoreductase SoxD), *tsdA* (thiosulfate dehydrogenase), *fccB* (sulfide dehydrogenase flavoprotein chain), *fccA* (cytochrome subunit of sulfide dehydrogenase), *sqr* (sulfide:quinone oxidoreductase), *soeA*, *soeB*, *soeC* (sulfite dehydrogenase (quinone) subunits SoeA, SoeB, and SoeC). Nitrogen cycling genes: *narG*, *narH*, *narI* (alpha, beta, and gamma subunit of nitrate reductase/nitrite oxidoreductase), *napA* (nitrate reductase (cytochrome)), *napB* (nitrate reductase (cytochrome), electron transfer subunit), *nrfH* (cytochrome c nitrite reductase small subunit), *nrfA* (nitrite reductase (cytochrome c-552)), *nirB* (nitrite reductase (NADH) large subunit), *nirD* (nitrite reductase (NADH) small subunit), *nirK* (nitrite reductase (NO forming). Phosphorus cycling genes: *phnG* (carbon-phosphorus lyase core complex subunit), *phnM* (alpha-D-ribose 1-methylphosphonate 5-triphosphate diphosphatase), and *phoX* (alkaline phosphatase). Cytochrome c oxidase genes: caa3-type cytochrome c oxidase (*coxABCD*). cbb3-type cytochrome c oxidase (*cooNOPQ* and *cooNQ*)

A total of 102 high-quality microbiome MAGs, including 49 AAP and 53 non-AAP MAGs, were recovered from the metagenomes of the global lake bloom aggregate samples. The MAGs were classified by GTDB-Tk [60], and about 75% of the AAP MAGs could be classified at the genera level. The majority of the AAP bacteria MAGs were classified [60] as Alphaproteobacteria (35 MAGs) and Betaproteobacteria (11 MAGs). Alphaproteobacteria AAP bacteria MAGs included *Aquidulcibacter* (5), *Rhizobium* (3), *Rhodobacter* (3), *Bosea* (2), *Elioraea* (2), *Phreatobacter* (2), *Roseomonas* (2), *Porphyrobacter* (1), and *Methylobacterium* (1) (Fig. 2A). Among Betaproteobacteria AAP bacteria, five MAGs were unidentified members of family of Burkholderiaceae. The remaining MAGs were not identified, and of these, most similar reference genomes in the GTDB were verified to be AAP bacteria based on the presence of genes encoding anoxygenic photosynthesis. The majority of the non-AAP bacteria MAGs were Bacteroidetes (15 MAGs), Alphaproteobacteria (13 MAGs), and Gammaproteobacteria (9 MAGs) (Fig. 2B).

### Metabolic pathways in the *Microcystis* interactome

**Fig. 3:**
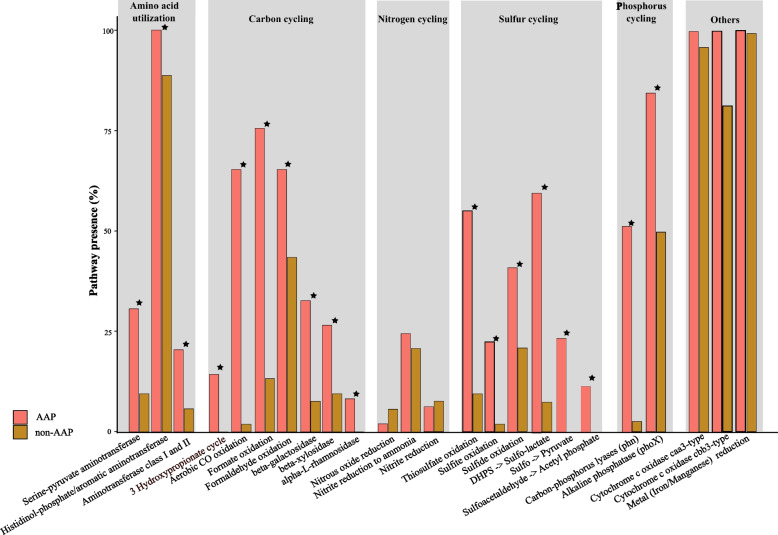
Pathway presence (represented as a percentage within the community) in AAP bacteria MAGs and non-AAP bacteria MAGs within *Microcystis* bloom samples of global lakes. Kolmogorov–Smirnov test, *p* < 0.05 was used to determine whether representation in genomes was significantly different. Stars indicated coverage of pathways was significantly different between AAP and non-AAP MAGs. Specific pathways were present based on key genes suggested by METABOLIC-C v4.0 [58]

The presence of *caa3* and *cbb3*-type cytochrome c oxidases in most MAGs indicated that abundant microbiome bacteria were mostly obligate or facultative aerobic bacteria. Of the 104 biochemical pathways identified in the microbiome genome database, 18 pathways occurred in significantly more AAP bacteria than non-AAP bacteria (Fig. 3; using the Kolmogorov–Smirnov test, *P* < 0.05). For example, the carbon monoxide (CO) oxidation pathway (*coxSML*) occurred in 31 of 49 AAP MAGs (63%) but in only one of 53 non-AAP MAGs (2%) (Fig. 2). More AAP bacteria had genes encoding pathways of amino acid utilization, oxidation of CO, formate, thiosulfate, sulfide, and sulfite, reduction of nitrate and nitrite to ammonia, DHPS (2,3-dihydroxypropane-1-sulfonate) catabolism, and organic P mineralization, while more non-AAP bacteria had genes encoding pathways of denitrification, including genes for reduction of nitric oxide and nitrous oxide (Fig. 3). AAP bacteria containing genes for oxidation of CO, formate, and reduced S are also capable of reducing nitrate and nitrite via ammonification. *coxSML* genes (oxidation of CO) and S oxidation genes encode aerobic enzyme complexes, while formate oxidation involves an anaerobic enzyme complex. These findings suggest that some AAP bacteria have versatile lifestyles, as they can carry out carbon monoxide assimilation and S oxidation in the presence of oxygen and formate oxidation with nitrate and iron as electron acceptors in the absence of oxygen. The most abundant AAP bacterium, *Roseomonas*, possessed genes encoding metabolic pathways for obtaining energy from organic carbon, CO, and sulfide oxidation, and sunlight via anoxygenic photosystems (Fig. 2A), as well as for being able to switch between aerobic respiration, anaerobic respiration, and fermentation.

### Metabolic gene abundances in the *Microcystis* interactome

**Fig. 4:**
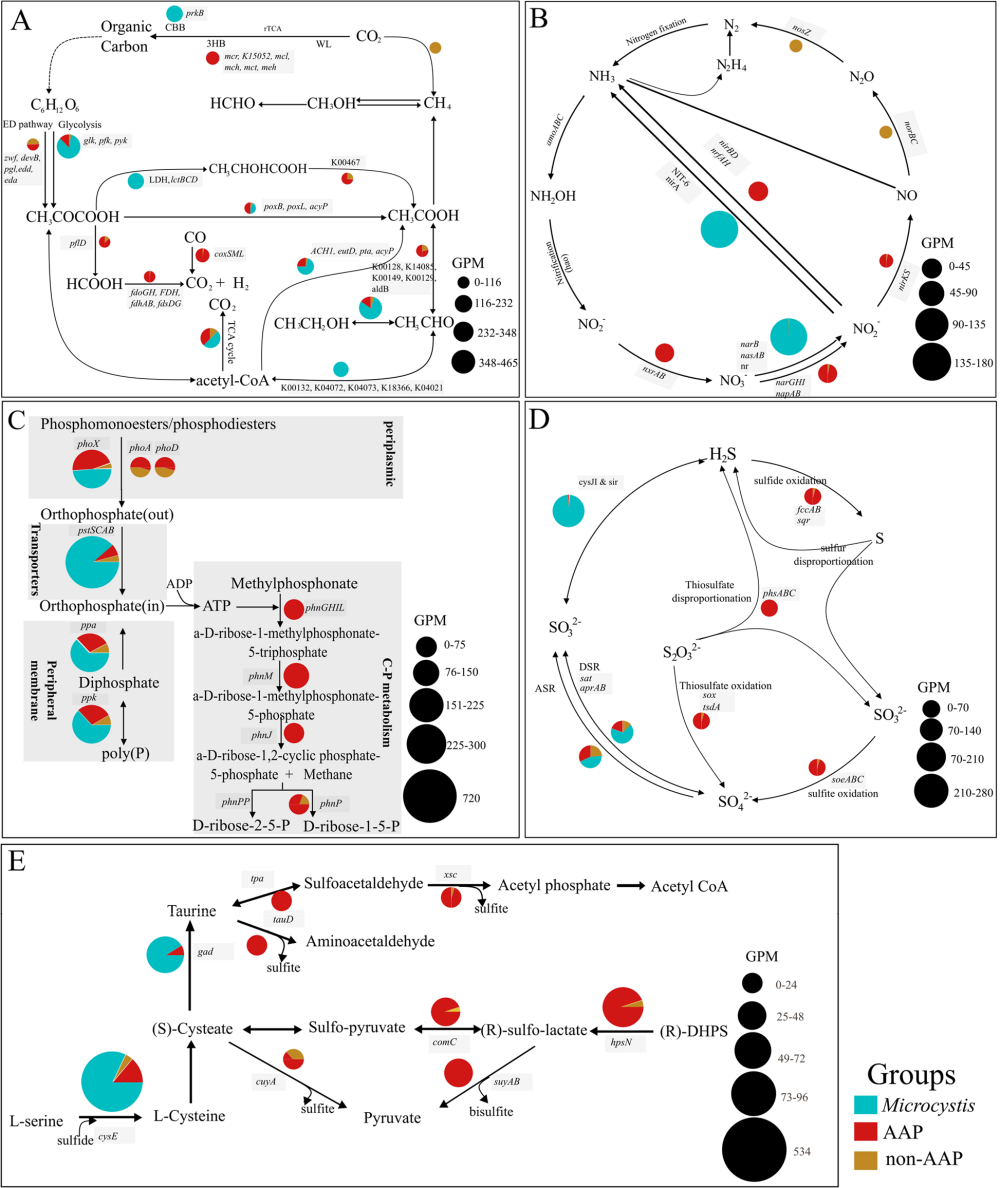
Biochemical pathways for microbial synthesis and catabolism of carbon (**A**), nitrogen (**B**), phosphorus (**C**), sulfur (**D**), and organic sulfur (**E**) metabolites in *Microcystis* (blue), AAP (red), and non-AAP (yellow) from global bloom samples. The genes per million reads (GPM) were calculated to understand the importance of each process in the biogeochemical cycles. The size of the pie chart in panels is proportional to the relative abundance of each gene involved in the pathway. rTCA, reversal citric acid cycle; CBB, Calvin–Benson–Basham; WL, Wood–Ljungdahl; ED, Entner-Doudoroff; 3HB, 3-hydroxypropionate bicycle. DSR, dissimilatory sulfate reduction; ASR, assimilatory sulfate reduction. Key genes involved in the pathways are shadowed gray. The key genes involved in conversion of acetaldehyde to ethanol and TCA cycles are described here (https://github.com/xuechunxu/DiTing/blob/master/Pathway_formulas.txt)

To aid in understanding the potential for interactions between *Microcystis* and associated bacteria, relative abundances (expressed as GPM) of metabolic genes in the 10 lake metagenomes were determined (Fig. 4). For each lake, 85.5 to 95.1% of microbiome bacteria reads were mapped onto the Aggregate Genome Database, indicating that the Aggregate Genome Database was representative of the microbiota present during blooms.

#### Carbon cycling

For CO_2_ fixation, *Microcystis* uses the Calvin–Benson–Bassham cycle (CBB), and AAP bacteria appear to use the 3-hydroxypropionate bicycle (3-HB). No reverse citric acid cycle or Wood–Ljungdahl cycles were identified (Fig. 4A). However, only five of six marker genes of the 3-HB cycle were detected in the communities, and the key gene encoding propionyl-CoA carboxylase was not detected, suggesting that 3-HB cycle was incomplete in the aggregate AAP bacteria. The presence of genes encoding L-lactate dehydrogenase and alcohol dehydrogenase in *Microcystis* genomes suggests that *Microcystis* can also produce lactic acid and ethanol during fermentation. A previous study has also documented the production of acetate and ethanol as fermentation products [78]. If produced, the fermentation products may be further utilized by associated bacteria. In global metagenomic data, genes involved in acetate/ethanol catabolism, including the genes encoding acetate kinase (AAP: 56.6 GPM vs non-AAP: 8.6 GPM), phosphotransacetylase (AAP: 49.1 GPM vs non-AAP: 4.2 GPM), and isocitrate lyase (AAP: 56.9 GPM vs non-AAP: 28.3 GPM) [79], were enriched in AAP bacterial communities. C1 metabolism pathways, including formate production and/or oxidization when anoxic conditions are present and CO oxidation under oxic conditions, were enriched in AAP bacteria (Fig. 4A). Formate metabolism was indicated by the presence of formate C-acetyltransferase (*pflD*), which catalyzes formate production during pyruvate degradation and formate dehydrogenase (*fdh/fdo*), catalyzing formate oxidation to CO_2_ and H_2_.

#### Nitrogen cycling

The dominant nitrogen cycling pathways were assimilatory nitrate and nitrite reduction in *Microcystis* (Fig. 4B). AAP bacteria were enriched in the dissimilatory nitrate reduction pathways, including nitrate reduction to nitrite and nitrate reduction to ammonium (DNRA). However, there is no correlation between the abundance of anoxygenic photosynthesis and the pathways involved in nitrogen cycling. This lack of a relationship may be because bacteria in some lakes (Aasee, FP23, and Villerest) had high relative abundances of anoxygenic photosynthesis genes but had none of the DNRA genes, including *narGHI*, *nirBD*, or *nrfAH* genes (Fig. S6). In those lakes, nitrate reduction (denitrification) pathways were primarily expressed by non-AAP bacteria. No genes encoding anammox, including *hzsABC* and *hdh*, were identified in the Aggregate Genome Database or contigs assembled from each metagenome.

#### Phosphorus cycling

*Microcystis* had the most abundant P utilization pathway genes, including orthophosphate transport (*pstSCAB*), polyphosphate synthesis (*ppK*), and hydrolysis (*ppA*). Phytoplankton are known to store inorganic P as polyphosphate (PolyP) in cells when P is abundant and break it down when P is limiting [80]. Three bacterial alkaline phosphatase families (*phoA*, *phoD*, and *phoX*) were identified, and over 48% of *phoX* gene reads, common among cyanobacteria [81], were present in *Microcystis*. However, *phoA* and *phoD* were only associated with AAP and non-AAP bacteria (Fig. 4C). Organophosphonate mineralization genes (*phnGHIL*, *phnJ*, and *phnM*) were mainly found in AAP bacteria (Fig. 4C). Interestingly, methane can be produced by C–P lyase complex (*phnJ*), indicating that AAP bacteria could also be involved in methane production.

#### Sulfur cycling

Assimilatory sulfate reduction (*cysJI*) and cysteine biosynthesis (*cysE*) were the most abundant genes involved in sulfur cycling observed in *Microcystis* and microbiome genomes, with over 98% of *cysJI* genes and 70% of *cysE* genes in the metagenome derived from *Microcystis* (Fig. 4DE). This indicates that *Microcystis* may be capable of assimilatory sulfate reduction to sulfide and incorporation of sulfide into the amino acids methionine and cysteine. The primary S-cycling function of associated bacteria appears to be S oxidation. AAP bacteria had genes relating to several oxidation processes, including sulfide (*fccAB*), thiosulfate (*sox*), and sulfite oxidation (*soeABC*). AAP bacteria were likely the dominant players in the catabolism of dissolved organic sulfur (DOS) metabolites. The sulfonate compound 2,3-dihydroxypropane-1-sulfonate (DHPS) is one of the most abundant organic sulfur compounds in the biosphere [82]. DHPS catabolase (*hpsN*) was the most abundant gene in the DOS catabolism pathway among AAP bacteria.

### AAP bacteria gene expression during nutrient cycling

**Fig. 5:**
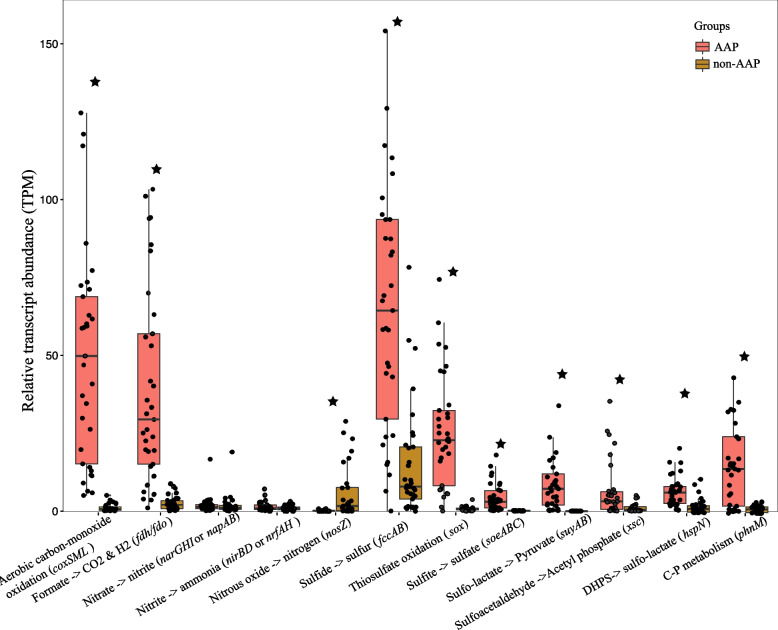
Relative transcript abundance of biogeochemical pathways in metatranscriptomic samples from Lakes Erie and Taihu (Table S2). The transcripts per million (TPM) was calculated. The interquartile range is represented within the box. The lower and upper hinge of the box represents the 25th and 75th percentiles, respectively. Stars indicate that the relative abundance of the pathways varied significantly between AAP and non-AAP groups (pairwise Wilcoxon test with *p*-values corrected with Benjamini–Hochberg FDR method). Key genes for each pathway are given. Metatranscriptomic data are publicly available from the NCBI SRA database, courtesy of [32, 74, 83], and the accession numbers and additional information about the metatranscriptomes are shown in Table S2

The relative expression data comparing AAP bacteria and non-AAP bacteria in Lake Erie and Taihu during blooms were generally similar to the corresponding relative abundances obtained by the metagenome-based community analysis. The metatranscriptomic analysis showed that *Microcystis* had predominantly assimilatory pathway transcriptional expression [32, 74, 83]. Relative to non-AAP bacteria, AAP bacteria produced relatively higher levels of transcripts for carbon (CO oxidation and formate oxidation), sulfur (sulfide, thiosulfate, and sulfite oxidation, DOS catabolism), and phosphorus metabolism (C-P metabolism), while non-AAP bacteria displayed higher levels of transcripts involved in nitrous oxide reductase (*nosZ*) (Fig. 5). In addition, both AAP bacteria and non-AAP bacteria had low transcript abundance for genes associated with nitrogen metabolism compared to the other nutrient metabolisms (Fig. 5).

**Fig. 6:**
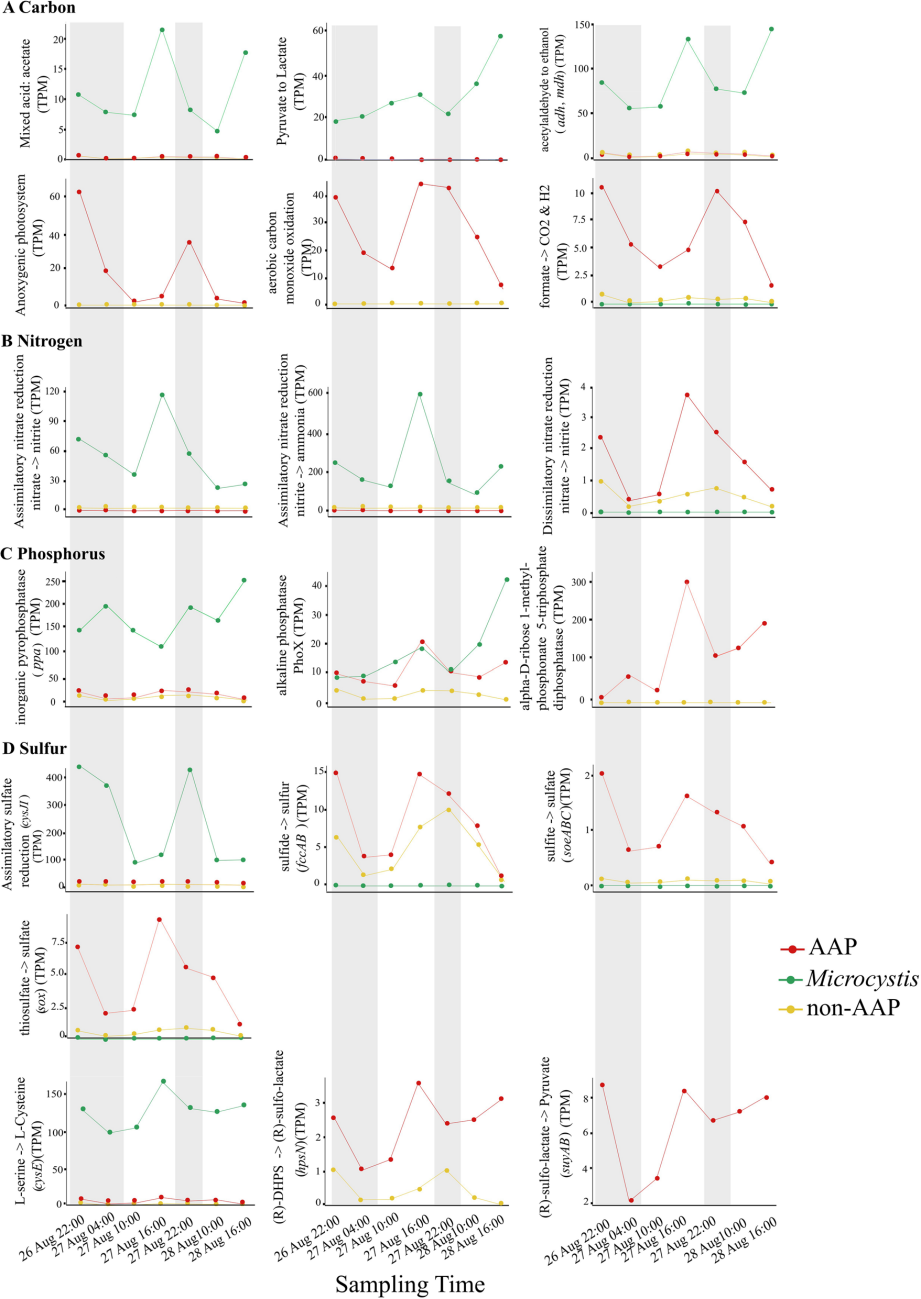
Diel transcriptional levels (TPM) of genes for microbial synthesis and catabolism of carbon (**A**), nitrogen (**B**), phosphorus (**C**), and sulfur (**D**) in *Microcystis *(green), AAP (red), and non-AAP (yellow). Gray shading indicates periods between sunset and sunrise. Samples were collected in western Lake Erie in late August 2014. For more detailed information about the samples, please refer to Table S4

Diel transcriptional patterns of biogeochemical pathway genes from *Microcystis*, AAP bacteria, and non-AAP bacteria in western Lake Erie during late August 2014 were also observed (Fig. 6). Transcripts involved in the production of ethanol, acetate, and lactate from *Microcystis* increased primarily during the day. The relative abundances of anoxygenic photosynthesis transcripts and formate oxidation transcripts from AAP bacteria increased primarily at night. Conversely, the aerobic carbon monoxide oxidation transcript abundance from AAP bacteria increased during the day, yielding maximum relative abundances at 16:00 h. Genes for the DNRA pathway showed higher relative transcript abundances during the day. Phosphorus acquisition and uptake (*pho*, *ppa*) by *Microcystis* were found to be relatively constant over the entire period. However, organophosphonate metabolism (*phnJ*) from AAP bacteria was highly upregulated during the day, and their transcriptional expression rapidly decreased at night. For sulfur metabolism, the assimilatory pathways (*cysJI* and *cysE*) were expressed by *Microcystis* and showed higher relative transcript abundances during the day*.* The assimilatory sulfate reduction pathway (*cysJI*) exhibited higher relative transcript abundances at night. Conversely, the biosynthesis of cysteine (*cysE*) displayed higher relative transcript abundances during the day. The genes involved in inorganic sulfur oxidation (*soeABC*, *sox*) and organic sulfur catabolism (*hpsN*, *suyAB*) were expressed almost exclusively by AAP bacteria, and their transcript abundances increased during the day. The *fccAB* gene involved in sulfide oxidation was expressed by both AAP bacteria and non-AAP bacteria in those samples, and their transcript abundances also increased during the day.

## Discussion

The microenvironment surrounding algae and algal colonies in which bacteria were often observed as being abundant was first termed the “phycosphere” by Bell and Mitchell [21]. Such associations between bacteria and bloom-forming algae, particularly cyanobacteria, are well-documented and have often been speculated as being symbiotic (e.g., [22, 84]) with the associated bacteria potentially representing a microbiome analogous to the microbiome concept described for humans [85], soils [86], and coral reefs [87]. The *Microcystis* phycosphere possesses unique characteristics. Firstly, the *Microcystis* aggregates shield inhabiting bacteria from grazers [14, 16], which is likely important as AAP bacteria are relatively large and therefore more susceptible to protist grazing than other smaller bacteria [88]. Due to intense top-down pressure, the number of AAP bacteria in waters is relatively low [89]. However, with the protection of the aggregates, we observed that the proportion of AAP bacteria in global bloom samples can exceed 20% of the non-cyanobacterial bacteria, whereas the proportion of AAP bacteria in surrounding water is usually less than 10% of the non-cyanobacterial community (Fig. S5). Secondly, *Microcystis* produces reduced organic nutrients that bacteria can utilize to gain energy. These reduced organics include a variety of organic sulfur metabolites [90], such as cysteine and DHPS, which could provide energy equivalent to, or greater than, organic carbon compounds during oxidative processes [91]. Comparative genomic analysis has shown that genes involved in DOS catabolism and oxidation of reduced sulfur metabolites are present in fast-growing AAP bacteria (Fig. 3). Thirdly, *Microcystis* creates a distinctive environment with large fluctuations in DO and pH (Fig. S3), which further selects for specific bacteria. Genes involved in respiratory and fermentative processes are ubiquitous in associated bacterial genomes (Figs. 2 and 3) and are highly responsive to the diel environmental fluctuations induced by *Microcystis* activities (Fig. 6). This observation suggests AAP bacteria in bloom aggregates can adjust to environmental fluctuations created by *Microcystis* activities by switching between aerobic and anaerobic processes. Lastly, *Microcystis* possesses gas vesicles that provide buoyancy to the aggregates and thus higher access to sunlight. This access may provide advantages to photoheterotrophs, such as AAP bacteria, containing bacteriochlorophyll with maximal absorption at ~ 870 nm. Bacteriochlorophyll-based photosynthesis under infrared light has been shown to significantly reduce respiration and enhance the assimilation of organic compounds by AAP bacteria [92]. Thus, *Microcystis* buoyancy may provide AAP bacteria a mechanism for maximizing photosynthesis while reducing respiration. Cook et al. [11] recently postulated that cyanobacteria-microbiome associations constitute complex interactomes (sensu [93]), consisting of one to several dominant cyanobacterial species and multiple bacterial taxa, which have coevolved to form a community of mutualistic and synergistic species, each with unique metabolic capabilities that are critical to the growth, maintenance, and demise of cyanobacterial blooms. As temperatures increase during the late spring and summer, *Microcystis* quickly forms a dense layer of biomass on the surface [1], leading to depletion of N and/or P [34]. As inorganic nutrients are depleted, *Microcystis* must rely on microbial partners to satisfy needs for essential nutrients. Knowledge of the biogeochemical interactions between *Microcystis* and its microbiome is key to understanding mechanisms that allow sustained growth throughout the season.

### AAP bacteria populations in the aggregates

Initial experiments looking at the ecology of the bloom showed a significantly decreased richness in the aggregate community relative to the free-living community (Fig. S4). This observation, which has been previously reported [9, 25], suggests that physical, chemical, or biological factors within the aggregates restrict or enrich the microbiota of specific taxonomic or functional groups. Beta-diversity measures also showed significant differences between aggregates and free-living samples for the weighted UniFrac metric (Fig. S4), suggesting substantial differences in community composition between the two groups.

The nine groups of AAP bacteria (confirmed by the presence of genes encoding anoxygenic photosynthesis in MAGs) that were enriched in *Microcystis* aggregates (Figs. 1 and 2) included two novel genera, suggesting further unknown diversity. Nevertheless, our finding that specific AAP bacteria were consistently present in high abundance across all global samples (Fig. S5) suggests the potential existence of a core functional microbiome comprised of AAP bacteria across *Microcystis* blooms, in line with the notion that a core *Microcystis* microbiome may not be defined at the species or genus level [23, 75].

### Biogeochemical interactions between *Microcystis* and AAP bacteria

During dark anoxic conditions and light/dark cycle, *Microcystis* was found to ferment stored sugar into ethanol, acetate, and lactate [78, 94, 95]. Transcripts involved in the production of ethanol, acetate, and lactate from *Microcystis* increased primarily during the day (Fig. 6), in line with the previous physiological study [95], indicating the presence of anoxic micro-niches within the bloom during the day. However, fermentation products, when they accumulate, can inhibit *Microcystis* growth [78, 96]. Genes involved in ethanol catabolism and formate oxidation were enriched in AAP bacterial communities, suggesting that AAP bacteria obtained energy by degrading and detoxifying the fermentation product. In addition, the enriched β-xylosidase and rhamnosidase observed in AAP bacterial genomes likely are involved in degradation of cyanobacterial EPS known to contain rhamnose and xylose [12]. Interestingly, an associated AAP bacterium, *Niveispirillum cyanobacteriorum*, produces β-galactosidase, a catabolic enzyme with potential functions in polysaccharide degradation and not present in the related non-AAP species *Niveispirillum fermenti* and *Niveispirillum irakense* [27], indicating further adaptation associated with living in the phycosphere.

The most abundant aerobic pathway enriched and expressed in AAP bacterial communities is for CO oxidization. *coxSML* genes transcribing proteins involved in CO oxidation are present in many AAP bacteria, including the *Roseobacter* group [97, 98]. A previous study indicated that AAP bacteria could use light and CO oxidation as complementary energy sources to better survive under severe energy limitations [99]. The origin of CO remains unclear.

*Microcystis* blooms are generally nitrogen (N) limited during summer months [34, 100] and the most abundant and most highly expressed genes involved in N cycling present in *Microcystis* encode nitrogen assimilatory pathways. Dissimilatory nitrate reduction to ammonium (DNRA) by AAP bacteria is therefore likely to be an important source of NH_4_ + -N for *Microcystis*. Under anoxic conditions, biologically available N can be removed from ecosystems through anaerobic ammonium oxidation (anammox) or denitrification, whereas DNRA acts to conserve N within the system. Anammox often occurs at the interface between surface water and sediment porewater and is limited to areas that are relatively low in labile carbon, which often is not the case for near-surface freshwater sediments that support high biological productivity [101]. Furthermore, our data analysis did not identify any genes associated with anammox. Denitrification is also likely an important process within blooms with denitrification rates as high as 392 μmol m^−2^ h^−1^ reported in Lake Taihu blooms at high TN concentrations (6.58 mg N L^−1^), a rate much higher than observed in the sediments [18]. Our data indicated that denitrification genes were encoded and expressed in the majority of non-cyanobacterial non-AAP bacteria. For DNRA to be favored over denitrification, a high level of electron donors is needed [102]. The higher abundances of genes mediating organic C decomposition and C1 and S compound oxidation in AAP bacteria provide support for this concept.

DOP is a major component of the P pool in aquatic ecosystems and includes phosphomonoesters (-C-O-P) and phosphonates (C-P) [103]. The most abundant P cycling genes derived from *Microcystis* encode dissolved inorganic phosphate (DIP) assimilation (*ppa* and *ppk*) and transporter (*pst*) genes. Although *Microcystis* alkaline phosphatase activity was detected [33], *Microcystis* growth is thought to be inhibited by high concentrations of DOP, indicating that *Microcystis* most likely cannot metabolize DOP present at high levels. Associated bacteria have various DOP transporters, carbon-phosphorus lyases, and alkaline phosphatases, likely degrading DOP and providing DIP to *Microcystis*. Based on the presence and expression of *phn* genes in AAP bacteria, they likely play a significant role in P mineralization and methane production in *Microcystis* aggregates. *phn* genes are responsible for the process of demethylation of methylphosphonates, which can occur under aerobic conditions [104].

*Microcystis* is known to produce reduced sulfur organic compounds, which can also be self-toxic [43]. The primary roles of AAP bacteria in sulfur metabolism appear to be in catabolism of organic sulfur compounds and oxidation of reduced inorganic sulfur compounds. DHPS degradation pathways were previously observed in marine AAP bacteria genomes [105]. Our analysis showed that DHPS metabolic pathways were also present, converting DHPS to sulfite and pyruvate, and were highly expressed in many freshwater AAP bacteria. Sulfite could be further oxidized by AAP bacteria encoding sulfite-oxidizing enzymes (*soeABC*). Although oxidation of reduced sulfur compounds is usually thought to be carried out by anaerobic phototrophic sulfur bacteria [106], AAP bacteria (which are aerobic) appear to be capable and likely important in several S oxidation processes. Strong diel fluctuations in DO within aggregates and high transcript abundances of genes involved in inorganic sulfur oxidation (*soeABC*, *sox*) and organic sulfur catabolism (*hpsN*, *suyAB*) during the day indicate that sulfur oxidizers were active under aerobic conditions. Many AAP bacteria can oxidize inorganic sulfur compounds growing lithotrophically under aerobic conditions [107]. The ability to oxidize thiosulfate appears to be especially widespread in AAP bacteria species [108].

## Conclusions

**Fig. 7:**
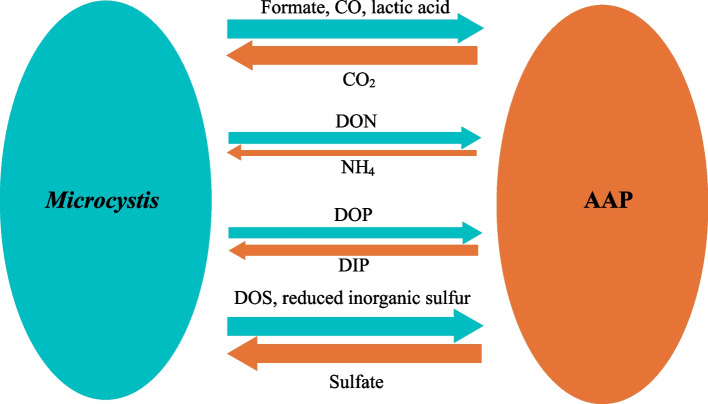
Schematic diagram of the biogeochemical network within *Microcystis* and AAP bacteria during bloom. The biogeochemical activities are displayed in a relative manner using different line thicknesses, based on the relative abundance of biochemical pathways from metagenomes shown in Fig. 4 and metatranscriptomes shown in Fig. 5

Based on the combined results of metagenomic and metatranscriptomic analyses, biogeochemical pathway reconstruction, and diel expressional analysis of *Microcystis* and its associated microbiome, we have proposed a biogeochemical network between *Microcystis* and the AAP bacteria-containing microbiome (Fig. 7). The network shows how the interactions between *Microcystis* and the microbiome can support the bloom when nutrients are limited. Metabolic dependencies can drive species co-occurrence in diverse microbial communities (sensu [109]), and cross-feeding may expand the metabolic niche of bacteria [110]. Our results can also help inform the development of new strategies for the mitigation of bloom events. For example, the sulfur-cycling-enabled mutualism between *Microcystis* and AAP bacteria highlights the importance of reducing S input as a potential strategy for the mitigation of bloom events, in addition to reducing N and P input. Elevated sulfate concentrations lead to increased sulfide production by *Microcystis* and phosphorous mobilization [111, 112]. This, in turn, promotes biomass formation and formation of reduced sulfur by *Microcystis* [90], providing ample reduced sulfur as an energy source for AAP bacteria and improving internal nutrient cycling within aggregates. However, while support for our hypotheses is derived from metagenomic and metatranscriptomic analyses, further investigation through metabolomic studies will be essential for testing the hypotheses regarding shared or complementary pathways. Additionally, the *Microcystis*-microbiome biogeochemical network is a complex interactome that will need much more study to unravel its complexity.

## Supplementary Information

**Additional file 1:** **Fig. S1.** Map of Lake Taihu and the sampling sites at Meiliang Bay and Zushan Bay, and Taihu Laboratory for Lake Ecosystem Research (TLLER). **Fig. S2.** Linear relationship between concentrations of DOC and Chl a of water samples in Lake Taihu. Data are based on 8 samples from site 1 at Meiliang Bay in September 2018. **Fig. S3.** Diel variation of DO and pH during a Microcystis bloom. Samples were collected from 51 site 1 in Meiliang Bay, Taihu, over a 24-h period from 10 to 10 AM on 10–11 August and 10–11 October 2018. Data are mean ± standard deviation (SD) (*n* = 3). **Fig. S4.** Alpha- and beta-diversity of samples from Lake Taihu. Free-living communities are displayed in blue and aggregate communities in orange. PD faith is used as an index for alpha diversity (A). Significant differences between the groups are indicated with asterisks (***p < 57 0.001). For beta-diversity (B), PCoA plot of the weighted UniFrac measures is shown. The x- and y-axes represent the first and second principal coordinates with the proportion of variance. Both diversity measures show significant differences between free-living and aggregate 60 communities. **Fig. S5.** Relative abundances of AAP bacterial genera in non-cyanobacterial communities in bloom samples from Lake Taihu (A) and ten global lakes (B). The sample IDs in Lake Taihu are shown in Table S1. **Fig. S6.** Relationships between gene abundance of anoxygenic photosystem pathways and important C, N, S, and P cycling pathways. Instead of using genes from MAGs, genes were derived from contigs co-assembled by metaSPAdes v3.15.4 using the metagenome data from each lake with Microcystis reads removed. Two biological replicates were obtained for each sample. Gene identification, annotation, and KO (KEGG Orthology) analysis were described in the text. Then reads of the ten lake metagenomes were mapped to genes derived from the contigs, and calculated GPMs. The GPMs were the input to calculate the relative abundance of pathways of each metagenome using formulae suggested by DiTing v0.9 [5]. **Table S1.** Collection date, location, sample type, and accession information for Lake Taihu 22 samples. **Table S2.** Source, location, date of collection and accession information for Lake Erie and Taihu transcriptome samples. **Table S3.** Taxonomic, completeness and contamination data for non-redundant microbiome MAGs. **Table S4.** Diel Bloom transcriptomes from Lake Erie. **Table S5.** Environmental variables of Meiliang Bay (M) and Zushan Bay (Z) in Lake Taihu. Chl a concentrations indicated that the 38 Microcystis bloom grew rapidly peaking between August and September, and the bloom began to decline in October. The pH in bloom 39 samples (from August to October) ranged from 8.0 to 10.0. The mass ratios of total nitrogen (TN) to total phosphorus (TP) ranged from 40 6.7 to > 31.8, but were lowest in the months of summer and fall, suggesting the Microcystis bloom was potentially N-limited.

## Data Availability

The raw sequencing data were submitted to the NCBI SRA database under the accession ID PRJNA985885. The genomes of AAP isolates in *Microcystis* aggregates from Lake Taihu were submitted to the NCBI SRA database under the accession ID PRJNA427797, PRJNA427794, PRJNA427795, PRJNA427804, PRJNA395960, and PRJNA382246.
