# Correction: Aerobic anoxygenic phototrophs play important roles in nutrient cycling within cyanobacterial Microcystis bloom microbiomes

**DOI:** 10.1186/s40168-025-02056-3

**Published:** 2025-02-15

**Authors:** Haiyuan Cai, Christopher J. McLimans, Helong Jiang, Feng Chen, Lee R. Krumholz, K. David Hambright

**Affiliations:** 1 School of Biological Sciences, University of Oklahoma, Norman, USA; 2 Nanjing Institute of Geography and Limnology, Chinese Academy of Sciences, Nanjing, China; 3 Institute of Marine and Environmental Technology, University of Maryland Center for Environmental Science, Baltimore, USA

**Correction** **:** **Microbiome 12, 88 (2024)**

**https://doi.org/10.1186/s40168-024-01801-4**

Following publication of the original article [1], the author reported that they missed including the following statement in the Funding section:

Funding in support of this research was also provided to K.D.H. by the US Geological Survey (Grant G21AP10181).

The original article has been updated.
