# Supplementary material for: Aerobic anoxygenic phototrophs play important roles in nutrient cycling within cyanobacterial Microcystis bloom microbiomes
Source: Microbiome. 2024 May 13;12:88. doi: 10.1186/s40168-024-01801-4 (PMC11089705; doi:10.1186/s40168-024-01801-4)
Supplement: Supplementary file 2 — Additional file 1: Fig. S1. Map of Lake Taihu and the sampling sites at Meiliang Bay and Zushan Bay, and Taihu Laboratory for Lake Ecosystem Research (TLLER). Fig. S2. Linear relationship between concentrations of DOC and Chl a of water samples in Lake Taihu. Data are based on 8 samples from site 1 at Meiliang Bay in September 2018. Fig. S3. Diel variation of DO and pH during a Microcystis bloom. Samples were collected from 51 site 1 in Meiliang Bay, Taihu, over a 24-h period from 10 to 10 AM on 10–11 August and 10–11 October 2018. Data are mean ± standard deviation (SD) (n = 3). Fig. S4. Alpha- and beta-diversity of samples from Lake Taihu. Free-living communities are displayed in blue and aggregate communities in orange. PD faith is used as an index for alpha diversity (A). Significant differences between the groups are indicated with asterisks (***p < 57 0.001). For beta-diversity (B), PCoA plot of the weighted UniFrac measures is shown. The x- and y-axes represent the first and second principal coordinates with the proportion of variance. Both diversity measures show significant differences between free-living and aggregate 60 communities. Fig. S5. Relative abundances of AAP bacterial genera in non-cyanobacterial communities in bloom samples from Lake Taihu (A) and ten global lakes (B). The sample IDs in Lake Taihu are shown in Table S1. Fig. S6. Relationships between gene abundance of anoxygenic photosystem pathways and important C, N, S, and P cycling pathways. Instead of using genes from MAGs, genes were derived from contigs co-assembled by metaSPAdes v3.15.4 using the metagenome data from each lake with Microcystis reads removed. Two biological replicates were obtained for each sample. Gene identification, annotation, and KO (KEGG Orthology) analysis were described in the text. Then reads of the ten lake metagenomes were mapped to genes derived from the contigs, and calculated GPMs. The GPMs were the input to calculate the relative abundance of pathways of e [file 40168_2024_1801_MOESM1_ESM.pdf]

Supplementary Materials for

Aerobic anoxygenic phototrophs play important roles in nutrient cycling within cyanobacterial *Microcystis* bloom microbiomes

Haiyuan Cai[1, 2], Christopher J. McLimans[2], Helong Jiang[1], Feng Chen[3], Lee R. Krumholz[2], and K. David Hambright[2*]

1. Nanjing Institute of Geography and Limnology, Chinese Academy of Sciences, Nanjing, China; 2. School of Biological Sciences, University of Oklahoma, Norman, USA; 3. Institute of Marine and Environmental Technology, University of Maryland Center for Environmental Science, Baltimore, USA .

*Corresponding author. Email: dhambright@ou.edu

SUPPLEMENTARY TABLES

Table S1. Collection date, location, sample type, and accession information for Lake Taihu samples.

Sample ID	Sample type	Location	Date	SRR accession
F-04-M	Free-living	Meiliang bay, Lake Taihu	Apr, 2018	SRR24980017
F-04-Z	Free-living	Zhushan Bay, Lake Taihu	Apr, 2018	SRR24980026
A-04-M	aggregates	Meiliang bay, Lake Taihu	Apr, 2018	SRR24980030
A-04-Z	aggregates	Zhushan Bay, Lake Taihu	Apr, 2018	SRR24980031
F-05-M	Free-living	Meiliang bay, Lake Taihu	May, 2018	SRR24980029
F-05-Z	Free-living	Zhushan Bay, Lake Taihu	May, 2018	SRR24980016
A-05-M	aggregates	Meiliang bay, Lake Taihu	May, 2018	SRR24980027
A-05-Z	aggregates	Zhushan Bay, Lake Taihu	May, 2018	SRR24980028
F-06-M	Free-living	Meiliang bay, Lake Taihu	Jun, 2018	SRR24980032
F-06-Z	Free-living	Zhushan Bay, Lake Taihu	Jun, 2018	SRR24980033
A-06-M	aggregates	Meiliang bay, Lake Taihu	Jun, 2018	SRR24980015
A-06-Z	aggregates	Zhushan Bay, Lake Taihu	Jun, 2018	SRR24980039
F-07-M	Free-living	Meiliang bay, Lake Taihu	Jul, 2018	SRR24980036
F-07-Z	Free-living	Zhushan Bay, Lake Taihu	Jul, 2018	SRR24980037
A-07-M	aggregates	Meiliang bay, Lake Taihu	Jul, 2018	SRR24980034
A-07-Z	aggregates	Zhushan Bay, Lake Taihu	Jul, 2018	SRR24980035
F-08-M	Free-living	Meiliang bay, Lake Taihu	Aug, 2018	SRR24980041
F-08-Z	Free-living	Zhushan Bay, Lake Taihu	Aug, 2018	SRR24980042
A-08-M	aggregates	Meiliang bay, Lake Taihu	Aug, 2018	SRR24980038
A-08-Z	aggregates	Zhushan Bay, Lake Taihu	Aug, 2018	SRR24980040
F-09-M	Free-living	Meiliang bay, Lake Taihu	Sep, 2018	SRR24980021
F-09-Z	Free-living	Zhushan Bay, Lake Taihu	Sep, 2018	SRR24980020
A-09-M	aggregates	Meiliang bay, Lake Taihu	Sep, 2018	SRR24980018
A-09-Z	aggregates	Zhushan Bay, Lake Taihu	Sep, 2018	SRR24980019
F-10-M	Free-living	Meiliang bay, Lake Taihu	Oct, 2018	SRR24980025
F-10-Z	Free-living	Zhushan Bay, Lake Taihu	Oct, 2018	SRR24980024
A-10-M	aggregates	Meiliang bay, Lake Taihu	Oct, 2018	SRR24980022
A-10-Z	aggregates	Zhushan Bay, Lake Taihu	Oct, 2018	SRR24980023

Table S2. Source, location, date of collection and accession information for Lake Erie and Taihu transcriptome samples.

Bioproject	Sample ID	Lake	Date	SRR accession	Submission by	reference
PRJNA262053	Station LET1 replicate 1	Lake Erie	Oct, 2013	SRR1596057	Stony Brook University	
PRJNA262053	Station LET1 replicate 2	Lake Erie	Oct, 2013	SRR1596599	Stony Brook University	
PRJNA262053	Station LET2 replicate 1	Lake Erie	Oct, 2013	SRR1596606	Stony Brook University	
PRJNA262053	Station LET2 replicate 2	Lake Erie	Oct, 2013	SRR1596607	Stony Brook University	
PRJNA262053	Station LET3 replicate 1	Lake Erie	Oct, 2013	SRR1601407	Stony Brook University	
PRJNA262053	Station LET3 replicate 2	Lake Erie	Oct, 2013	SRR1601408	Stony Brook University	
PRJNA262053	Station LET4 replicate 1	Lake Erie	Oct, 2013	SRR1601409	Stony Brook University	
PRJNA262053	Station LET4 replicate 2	Lake Erie	Oct, 2013	SRR1601410	Stony Brook University	
PRJNA262053	Station LET5 replicate 1	Lake Erie	Oct, 2013	SRR1601412	Stony Brook University	
PRJNA262053	Station LET5 replicate 2	Lake Erie	Oct, 2013	SRR1601413	Stony Brook University	
PRJNA262053	Station LET6 replicate 1	Lake Erie	Oct, 2013	SRR1601414	Stony Brook University	
PRJNA262053	Station LET6 replicate 2	Lake Erie	Oct, 2013	SRR1601415	Stony Brook University	
PRJNA262053	Station LET7 replicate 1	Lake Erie	Oct, 2013	SRR1601416	Stony Brook University	
PRJNA262053	Station LET7 replicate 2	Lake Erie	Oct, 2013	SRR1601417	Stony Brook University	
PRJNA354726	W4b	Lake Erie	Aug, 2014	SRR5079666	University of Tennessee Knoxville	

PRJNA354726	W13b	Lake Erie	Aug, 2014	SRR5079667	University of Tennessee Knoxville
PRJNA354726	W2b	Lake Erie	Aug, 2014	SRR5079668	University of Tennessee Knoxville
PRJNA354726	73 July A	Lake Erie	July, 2014	SRR5079670	University of Tennessee Knoxville
PRJNA354726	73 July B	Lake Erie	July, 2014	SRR5079671	University of Tennessee Knoxville
PRJNA354726	LE Aug1	Lake Erie	Aug, 2014	SRR5079672	University of Tennessee Knoxville
PRJNA354726	LE W13a	Lake Erie	Aug, 2014	SRR5079673	University of Tennessee Knoxville
PRJNA354726	Aug2b	Lake Erie	Aug, 2014	SRR5079674	University of Tennessee Knoxville
PRJNA354726	LE W4a	Lake Erie	Aug, 2014	SRR5079675	University of Tennessee Knoxville
PRJNA354726	LE W2a	Lake Erie	Aug, 2014	SRR5079676	University of Tennessee Knoxville
PRJNA354726	LE W6	Lake Erie	Aug, 2014	SRR5079677	University of Tennessee Knoxville
PRJNA354726	LE W8	Lake Erie	Aug, 2014	SRR5079678	University of Tennessee Knoxville
PRJNA354726	LE site Aug1	Lake Erie	Aug, 2014	SRR5079679	University of Tennessee Knoxville
PRJNA354726	LE Aug2a	Lake Erie	Aug, 2014	SRR5079681	University of Tennessee Knoxville
PRJNA823389	Time 48h Ammonium, replicate A	Lake Erie	Jul, 2019	SRR18613350	University of Tennessee
PRJNA823389	Time 48h Ammonium, replicate B	Lake Erie	Jul, 2019	SRR18613349	University of Tennessee
PRJNA823389	Time 48h Ammonium, replicate C	Lake Erie	Jul, 2019	SRR18613348	University of Tennessee
PRJNA823389	Time 48h Phosphate, replicate	Lake Erie	Jul, 2019	SRR18613357	University of

	A				Tennessee	
PRJNA823389	Time 48h Phosphate, replicate B	Lake Erie	Jul, 2019	SRR18613356	University of Tennessee	
PRJNA823389	Time 48h Phosphate, replicate C	Lake Erie	Jul, 2019	SRR18613354	University of Tennessee	
PRJNA823389	Time 48h Urea, replicate A	Lake Erie	Jul, 2019	SRR18613361	University of Tennessee	
PRJNA823389	Time 48h Urea, replicate B	Lake Erie	Jul, 2019	SRR18613360	University of Tennessee	
PRJNA823389	Time 48h Urea, replicate C	Lake Erie	Jul, 2019	SRR18613359	University of Tennessee	
PRJNA823389	Time 48h Nitrate, replicate A	Lake Erie	Jul, 2019	SRR18613365	University of Tennessee	
PRJNA823389	Time 48h Nitrate, replicate B	Lake Erie	Jul, 2019	SRR18613364	University of Tennessee	
PRJNA823389	Time 48h Nitrate, replicate C	Lake Erie	Jul, 2019	SRR18613363	University of Tennessee	
PRJNA823389	Time 48h Control, replicate A	Lake Erie	Jul, 2019	SRR18613353	University of Tennessee	
PRJNA823389	Time 48h Control, replicate B	Lake Erie	Jul, 2019	SRR18613352	University of Tennessee	
PRJNA823389	Time 48h Control, replicate C	Lake Erie	Jul, 2019	SRR18613351	University of Tennessee	
PRJNA823389	Time 0h Control, replicate A	Lake Erie	Jul, 2019	SRR18613367	University of Tennessee	
PRJNA823389	Time 0h Control, replicate B	Lake Erie	Jul, 2019	SRR18613366	University of Tennessee	
PRJNA823389	Time 0h Control, replicate C	Lake Erie	Jul, 2019	SRR18613355	University of Tennessee	
PRJNA359157	Cyanobacterial aggregates_Metatranscriptome	Lake Taihu	May, 2015	SRR5134202	Southeast University, China	[1]
PRJNA664620	CA151123	Lake Taihu	Nov, 2015	SRR12686996	Southeast University, China	[2]

PRJNA664620	CA151028	Lake Taihu	Oct, 2015	SRR12686983	Tsinghua University, China	[2]
PRJNA664620	CA150902	Lake Taihu	Sep, 2015	SRR12686984	Tsinghua University ,China	[2]
PRJNA664620	CA150806	Lake Taihu	Aug, 2015	SRR12686985	Tsinghua University, China	[2]
PRJNA664620	CA150804	Lake Taihu	Aug, 2015	SRR12686986	Tsinghua University, China	[2]
PRJNA664620	CA150701	Lake Taihu	Jul, 2015	SRR12686987	Tsinghua University, China	[2]
PRJNA664620	CA150615	Lake Taihu	Jun, 2015	SRR12686988	Tsinghua University, China	[2]
PRJNA664620	CA150608	Lake Taihu	Jun, 2015	SRR12686989	Tsinghua University, China	[2]
mgp103977*	Jul2016	Lake Taihu	July, 2016	4773250.3	Chinese Academy of Sciences, China	[3]
mgp103977*	Aug2016	Lake Taihu	Aug, 2016	4729510.3	Chinese Academy of Sciences, China	[3]
mgp103977*	Sep2016	Lake Taihu	Sep, 2016	4775083.3	Chinese Academy of Sciences, China	[3]
mgp103977*	Oct2016	Lake Taihu	Oct, 2016	4747924.3	Chinese Academy of Sciences, China	[3]

*Metatranscriptomes are publicly available from the MG-RAST server.

Table S3. Taxonomic, completeness and contamination data for non-redundant microbiome MAGs

accession	lineage	AAP bacteria YES or NO	Completeness (%)	Contamination (%)
JAJTDF01	*Planctomycetaceae* bacterium	NO	95.45	0
JAJTHL01	Alphaproteobacteria bacterium	NO	95.7	0
JAJTHN01	*Phycisphaeraceae* bacterium	NO	96.59	0
JADBXD01	*Chitinophagaceae* bacterium	NO	96.88	0
DA04	*Methylophilus sp.*	NO	96.89	0
JADDKF01	*Cutibacterium sp.*	NO	97.7	0
JADCBM01	*Methylobacterium sp.*	YES	97.75	0
JADBXC01	*Chitinophagaceae* bacterium	NO	97.78	0
JADBXP01	*Chitinophagaceae* bacterium	NO	97.78	0
JADCCD01	*Methylobacterium sp.*	YES	98.07	0
JADCGS01	*Burkholderiales* bacterium	YES	98.15	0
JADCGV01	*Burkholderiales* bacterium	YES	98.15	0
JADCCF01	*Methylobacterium sp.*	YES	98.36	0
TW01	*Flavobacterium sp.*	NO	98.44	0
JADCFT01	*Roseomonas sp.*	YES	98.51	0
SGWC01	*Exiguobacterium sp.*	NO	98.68	0
SGWE01	*Deinococcus sp.*	NO	99.15	0
DW08	*Flavobacterium sp.*	NO	99.28	0
DF15	*Brevundimonas sp.*	NO	99.35	0
JADBXR01	*Chitinophagaceae* bacterium	NO	99.51	0
TW09	*Flavihumibacter sp.*	NO	99.75	0
JADCGE01	*Roseomonas sp.*	YES	99.75	0
JAJTHB01	*Rickettsiales* bacterium	NO	100	0
JADCGF01	*Roseomonas sp.*	YES	100	0
JADCDU01	*Rhodobacter sp.*	YES	99.64	0.02
JADCHI01	*Burkholderiales* bacterium	NO	99.57	0.03
JAJTDL01	*Burkholderiaceae* bacterium	YES	99.03	0.08
JAJTIA01	Candidatus *Jidaibacter sp.*	NO	98.9	0.1
JADCDS01	*Rhodobacter sp.*	YES	99.33	0.1
JADCHW01	*Rhodocyclaceae* bacterium	YES	99.53	0.12
JADCEC01	*Rhodobacter sp.*	YES	99.94	0.12
JADCER01	*Roseomonas sp.*	YES	99.5	0.17
SGWF01	*Paenibacillus sp.*	NO	98.39	0.2
JADCDI01	*Rhodobacter sp.*	YES	99.64	0.2

JAJTEW01	*Comamonadaceae* bacterium	NO	99.6	0.22
JAJTDM01	*Rubrivivax sp.*	YES	95.35	0.23
JADCGR01	*Curvibacter sp.*	YES	97.51	0.23
JADCIV01	*Rhodocyclaceae* bacterium	YES	97.06	0.24
JADCEO01	*Rhodobacter* sp.	YES	98.83	0.25
TF07	*Sphingopyxis sp.*	YES	98.65	0.26
JADCIX01	*Rhodocyclaceae* bacterium	YES	98.58	0.29
JADCCN01	*Methylobacterium sp.*	YES	96.66	0.31
JADCCC01	*Methylobacterium sp.*	YES	98.22	0.31
JADCAS01	*Phenylobacterium sp.*	NO	97.62	0.32
JADCCL01	*Methylobacterium sp.*	YES	98.1	0.32
SGWD01	*Enterobacter sp.*	NO	99.89	0.35
DW05	*Sphingomonadaceae* bacterium	YES	98.12	0.39
JADBXZ01	*Bacteroidetes* bacterium	NO	99.05	0.41
JAJTEM01	*Burkholderiales* bacterium	NO	97.55	0.43
JADCDB01	*Aestuariivirga sp.*	YES	98.26	0.43
JADCGL01	*Telmatospirillum sp.*	YES	100	0.43
JAJTEG01	*Rhodobacter sp.*	YES	97.24	0.45
DF16	*Limnobacter sp.*	NO	98.36	0.45
JADCEN01	*Rhodobacter sp.*	YES	98.99	0.45
JADCHP01	*Burkholderiales* bacterium	NO	99.57	0.45
TF06	*Rhizobium/Agrobacterium sp.*	YES	98.91	0.46
DF14	*Silanimonas sp.*	NO	99.22	0.46
JAJTEN01	*Sphingobacteriales* bacterium	NO	98.14	0.48
DF05	*Hyphomonadaceae* bacterium	YES	96.37	0.49
JADCFX01	*Roseomonas sp.*	YES	98.73	0.5
JADCEV01	*Roseomonas sp.*	YES	99	0.5
JADCFA01	*Roseomonas sp.*	YES	99	0.5
JADCGA01	*Roseomonas sp.*	YES	99.25	0.5
TA06	*Rhizobium/Agrobacterium sp.*	YES	99.48	0.52
JADBZH01	*Phenylobacterium sp.*	NO	97.21	0.54
JADDJO01	*Burkholderia sp.*	NO	97.18	0.55
JAJTIE01	*Bacteroidetes* bacterium	NO	97.96	0.55
JADBYM01	*Cytophagales* bacterium	NO	97.45	0.6
JAJTDW01	Proteobacteria bacterium	NO	95.02	0.61
JADCCU01	*Methylocystis sp.*	YES	97.49	0.63

JADBYT01	*Bacteroidetes* bacterium	NO	99.52	0.63
JADBZC01	*Phenylobacterium sp.*	NO	97.13	0.65
TF08	*Brevundimonas sp.*	NO	99.59	0.65
JADCIZ01	*Rhodocyclaceae* bacterium	YES	95.73	0.66
DA12	*Pseudomonas sp.*	NO	98.15	0.76
JADCEM01	*Rhodobacter sp.*	YES	99.03	0.83
JADBZP01	*Phenylobacterium sp.*	NO	96.3	0.87
JAJTIC01	Proteobacteria bacterium	NO	95.98	0.89
JADBYQ01	*Cytophagales* bacterium	NO	97.45	0.89
JADCBK01	*Aquidulcibacter sp.*	YES	97.19	0.92
JADCGP01	*Rubrivivax sp.*	YES	98.83	0.93
JADCHQ01	*Rhodocyclaceae* bacterium	YES	97.79	0.95
JADBXW01	*Chitinophagaceae* bacterium	NO	96.31	0.99
JAJTHQ01	*Sediminibacterium sp.*	NO	96.8	0.99
JAJTHM01	*Sphingobacteriales* bacterium	NO	97.29	0.99
JADCHA01	*Cupriavidus sp.*	NO	99.03	0.99
JADCFN01	*Roseomonas sp.*	YES	99.5	1
JADCGC01	*Roseomonas sp.*	YES	99.75	1
JADCFQ01	*Roseomonas sp.*	YES	100	1
JADCAZ01	*Phenylobacterium sp.*	NO	97.94	1.01
JAJTER01	*Saprospiraceae* bacterium	NO	98.48	1.01
JADCHE01	*Burkholderiales* bacterium	YES	96.3	1.03
JAJTHY01	Candidatus *Jidaibacter* sp.	NO	96.7	1.1
TW08	*Pseudomonas sp.*	NO	99.25	1.1
JAJTDE01	*Rubrivivax sp.*	YES	95.99	1.11
JADCJE01	*Phycisphaerales* bacterium	NO	97.73	1.14
JAJTHT01	Betaproteobacteria bacterium	YES	96.68	1.16
JADCFP01	*Roseomonas sp.*	YES	98.42	1.16
CP035951	*Acidovorax sp.*	NO	98.97	1.16
JAJTFC01	*Planctomycetaceae* bacterium	NO	95.22	1.18
JADCJC01	*Rhodocyclaceae* bacterium	YES	96.45	1.18
JADCAL01	*Phenylobacterium sp.*	NO	97.94	1.2
JADCCY01	*Bradyrhizobium sp.*	NO	97.07	1.23
JADCBF01	*Phenylobacterium sp.*	NO	97.46	1.23
JAJTGP01	*Hyphomonadaceae* bacterium	YES	95.08	1.28
JAJTGY01	*Flammeovirgaceae* bacterium	NO	97.17	1.29
JADCAH01	*Phenylobacterium sp.*	NO	96.54	1.32

JAJTEA01	Betaproteobacteria bacterium	YES	95.73	1.37
JADCAP01	*Phenylobacterium sp.*	NO	97.94	1.43
JAJTHZ01	*Xanthomonadaceae* bacterium	YES	96.84	1.47
JADCFK01	*Roseomonas sp.*	YES	95.6	1.64
JAJTHH01	*Flammeovirgaceae* bacterium	NO	96.26	1.79
DF12	*Xanthomonadaceae* bacterium	NO	97.8	1.8
JAJTHS01	*Burkholderiales* bacterium	NO	95.11	2.03
JAINDL01	*Bryobacteraceae* bacterium	NO	98.26	2.17
JAJTHG01	*Gemmatimonadetes* bacterium	YES	96.15	2.2
JADCGQ01	*Rubrivivax sp.*	YES	97.2	2.34
DA05	*Hyphomonadaceae* bacterium	YES	97.19	2.38
JADCFW01	*Roseomonas sp.*	YES	99.75	2.49
JADCGK01	*Azospirillum sp.*	YES	98.91	2.55
TW06	*Rhizobium/Agrobacterium sp.*	YES	99.1	2.71
JAJTEJ01	*Rickettsiaceae* bacterium	NO	95.34	2.84

Table S4. Diel Bloom transcriptomes from Lake Erie.

Sample ID	Lake	Sampling time	SRR accession	reference
Diel_1S_2200h	western Lake Erie	26 Aug, 2014. 22:00h	SRR6048562	[4]
Diel_2S_0400h	western Lake Erie	27 Aug, 2014. 04:00h	SRR6466459	[4]
Diel_3S_1000h	western Lake Erie	27 Aug, 2014. 10:00h	SRR6466487	[4]
Diel_4S_1600h	western Lake Erie	27 Aug, 2014. 16:00h	SRR6048585	[4]
Diel_5S_2200h	western Lake Erie	27 Aug, 2014. 22:00h	SRR6048582	[4]
Diel_6S_1000h	western Lake Erie	28 Aug, 2014. 10:00h	SRR6048648	[4]
Diel_7S_1600h	western Lake Erie	28 Aug, 2014. 16:00h	SRR6466476	[4]

Table S5. Environmental variables of Meiliang Bay (M) and Zushan Bay (Z) in Lake Taihu. Chl *a* concentrations indicated that the *Microcystis* bloom grew rapidly peaking between August and September, and the bloom began to decline in October. The pH in bloom samples (from August to October) ranged from 8.0 to 10.0. The mass ratios of total nitrogen (TN) to total phosphorus (TP) ranged from 6.7 to >31.8, but were lowest in the months of summer and fall, suggesting the *Microcystis* bloom was potentially N-limited.

	**Temp (°C)**	**pH**	**TN (mg/L)**	**NO_3 (mg/L)**	**NH_4 (mg/L)**	**TP (mg/L)**	**PO_4 (mg/L)**	**N/P**	**Chl *a* (μg/L)**
Apr-M	13.2	7.3	3.82	1.03	0.27	0.12	0.01	30.2	6.0
May-M	20.2	7.5	2.84	1.26	0.18	0.10	0.01	27.9	6.4
Jun-M	24.6	7.8	2.59	0.65	0.25	0.10	0.02	24.5	14.9
Jul-M	27.2	8.2	2.73	0.44	0.50	0.20	0.02	13.5	23.4
Aug-M	29.0	9.5	2.20	0.10	0.31	0.35	0.05	6.1	79.8
Sep-M	27.6	9.2	2.54	0.11	0.18	0.30	0.08	8.3	63.6
Oct-M	19.6	8.7	4.08	2.55	0.50	0.20	0.08	19.8	43.1
Apr-Z	13.3	7.1	5.07	1.36	0.45	0.14	0.05	34.8	12.9
May-Z	21.3	7.2	4.42	1.33	0.42	0.15	0.07	29.1	23.4
Jun-Z	24.9	7.6	4.08	1.72	1.28	0.16	0.10	24.3	34.6
Jul-Z	27.4	8.4	3.69	0.52	0.27	0.23	0.14	16.0	37.7
Aug-Z	29.3	9.1	2.23	0.27	0.87	0.40	0.22	5.5	92.1
Sep-Z	27.5	8.6	3.03	0.70	1.02	0.25	0.09	12.4	69.8
Oct-Z	19.6	7.2	3.20	0.38	0.51	0.15	0.09	21.5	43.6

SUPPLEMENTARY FIGURES

Figure Legends

Fig. S1. Map of Lake Taihu and the sampling sites at Meiliang Bay and Zushan Bay, and Taihu Laboratory for Lake Ecosystem Research (TLLER).

Fig. S2. Linear relationship between concentrations of DOC and Chl *a* of water samples in Lake Taihu. Data are based on 8 samples from site 1 at Meiliang Bay in September 2018.

Fig. S3. Diel variation of DO and pH during a Microcystis bloom. Samples were collected from site 1 in Meiliang Bay, Taihu, over a 24-hour period from 10 AM to 10 AM on 10-11 August and 10-11 October 2018. Data are mean ± standard deviation (SD) ($n = 3$).

Fig. S4. Alpha- and beta-diversity of samples from Lake Taihu. Free-living communities are displayed in blue and aggregate communities in orange. PD faith is used as an index for alpha-diversity (A). Significant differences between the groups are indicated with asterisks (***$p < 0.001$). For beta-diversity (B), PCoA plot of the weighted UniFrac measures is shown. The x- and y-axes represent the first and second principal coordinates with the proportion of variance. Both diversity measures show significant differences between free-living and aggregate communities.

Fig. S5. Relative abundances of AAP bacterial genera in non-cyanobacterial communities in bloom samples from Lake Taihu (A) and ten global lakes (B). The sample IDs in Lake Taihu are shown in Table S1.

Fig. S6. Relationships between gene abundance of anoxygenic photosystem pathways and important C, N, S, and P cycling pathways. Instead of using genes from MAGs, genes were derived from contigs co-assembled by metaSPAdes v3.15.4 using the metagenome data from each lake with *Microcystis* reads removed. Two biological replicates were obtained for each sample. Gene identification, annotation, and KO (KEGG Orthology) analysis were described in the text. Then reads of the ten lake metagenomes were mapped to genes derived from the contigs, and calculated GPMs. The GPMs were the input to calculate the relative abundance of pathways of each metagenome using formulae suggested by DiTing v0.9 [5].

REFERENCES

1. Chen Z, Zhang J, Li R, Tian F, Shen Y, Xie X, Ge Q, Lu ZH. Metatranscriptomics analysis of cyanobacterial aggregates during cyanobacterial bloom period in Lake Taihu, China. Environ Sci Pollut Res Int. 2018;25:4811-25.
2. Zhu C, Zhang J, Wang X, Yang Y, Chen N, Lu Z, Ge Q, Jiang R, Zhang X, Yang Y, et al. Responses of cyanobacterial aggregate microbial communities to algal blooms. Water Res. 2021;196:117014.
3. Shi L, Cai Y, Gao S, Zhang M, Chen F, Shi X, Yu Y, Lu Y, Wu QL. Gene expression pattern of microbes associated with large cyanobacterial colonies for a whole year in Lake Taihu. Water Res. 2022;223:118958.
4. Davenport EJ, Neudeck MJ, Matson PG, Bullerjahn GS, Davis TW, Wilhelm SW, Denney MK, Krausfeldt LE, Stough JMA, Meyer KA, et al. Metatranscriptomic Analyses of Diel Metabolic Functions During a Microcystis Bloom in Western Lake Erie (United States). Front Microbiol. 2019;10.
5. Xue C, Lin H, Zhu X, Liu J, Zhang Y, Rowley G, Todd JD, Li M, Zhang X. DiTing: A Pipeline to Infer and Compare Biogeochemical Pathways From Metagenomic and Metatranscriptomic Data. Front Microbiol. 2021;12.

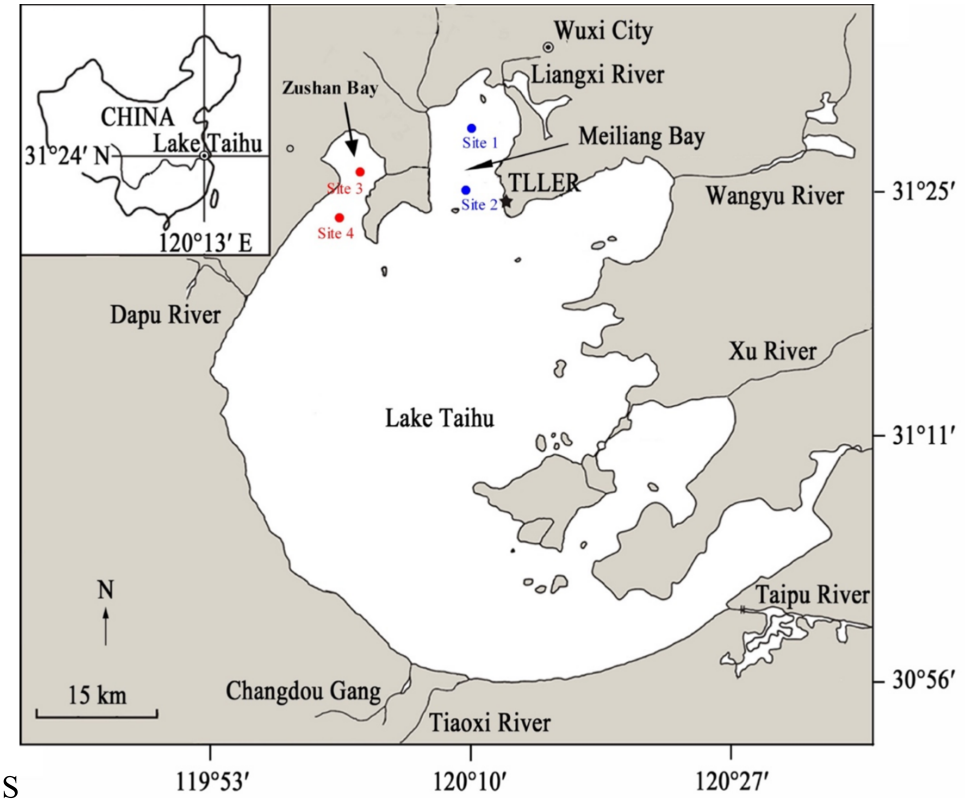

S

Figure S1

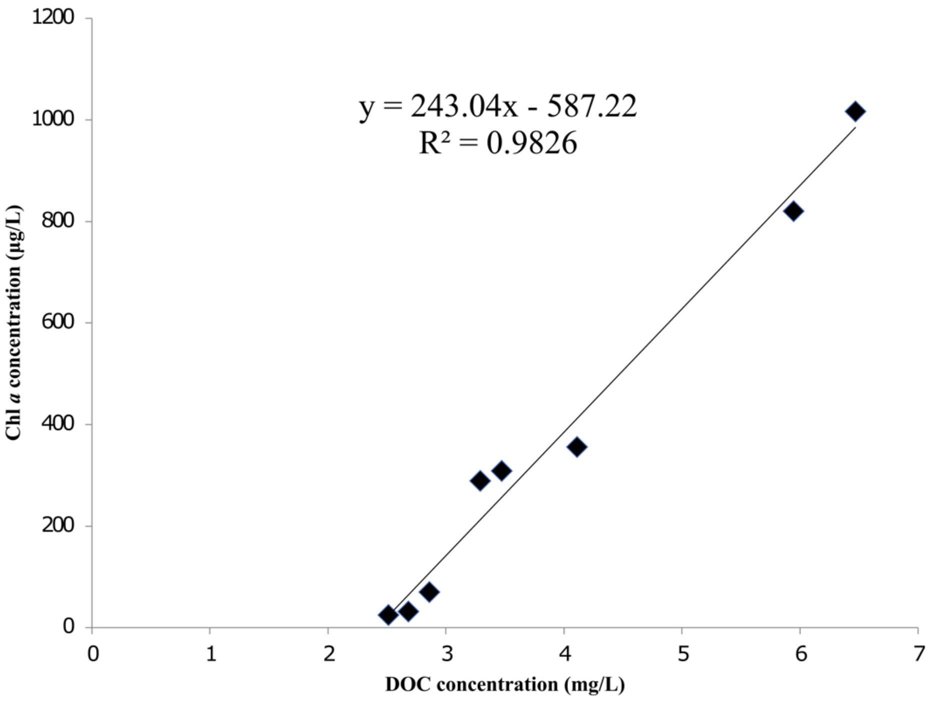

Figure S2

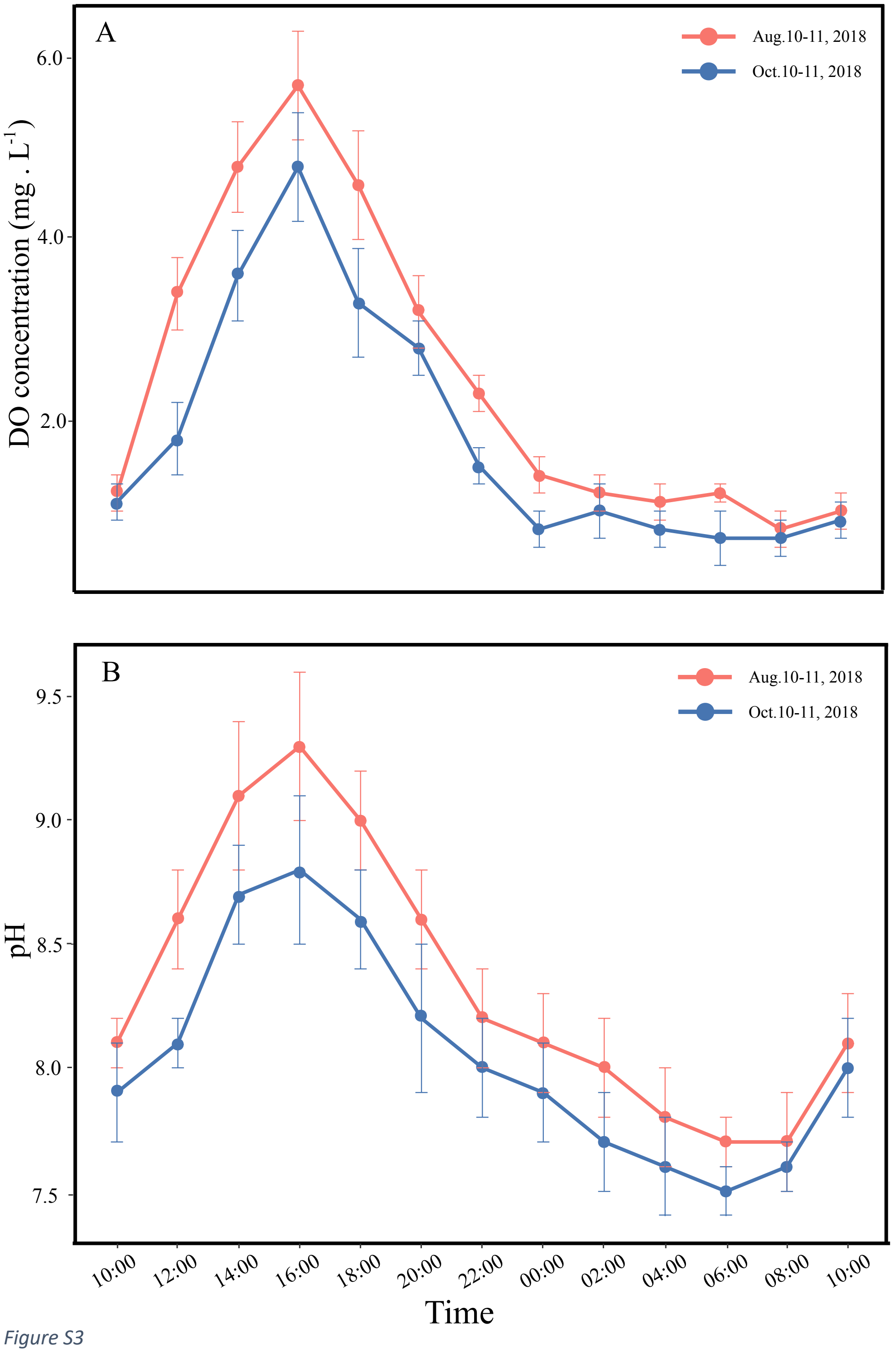
A
Aug.10-11, 2018
Oct.10-11, 2018
6.0
4.0
2.0
DO concentration (mg . L-1)
B
Aug.10-11, 2018
Oct.10-11, 2018
9.5
9.0
8.5
8.0
7.5
pH
10:00
12:00
14:00
16:00
18:00
20:00
22:00
00:00
02:00
04:00
06:00
08:00
10:00
Time

Figure S3

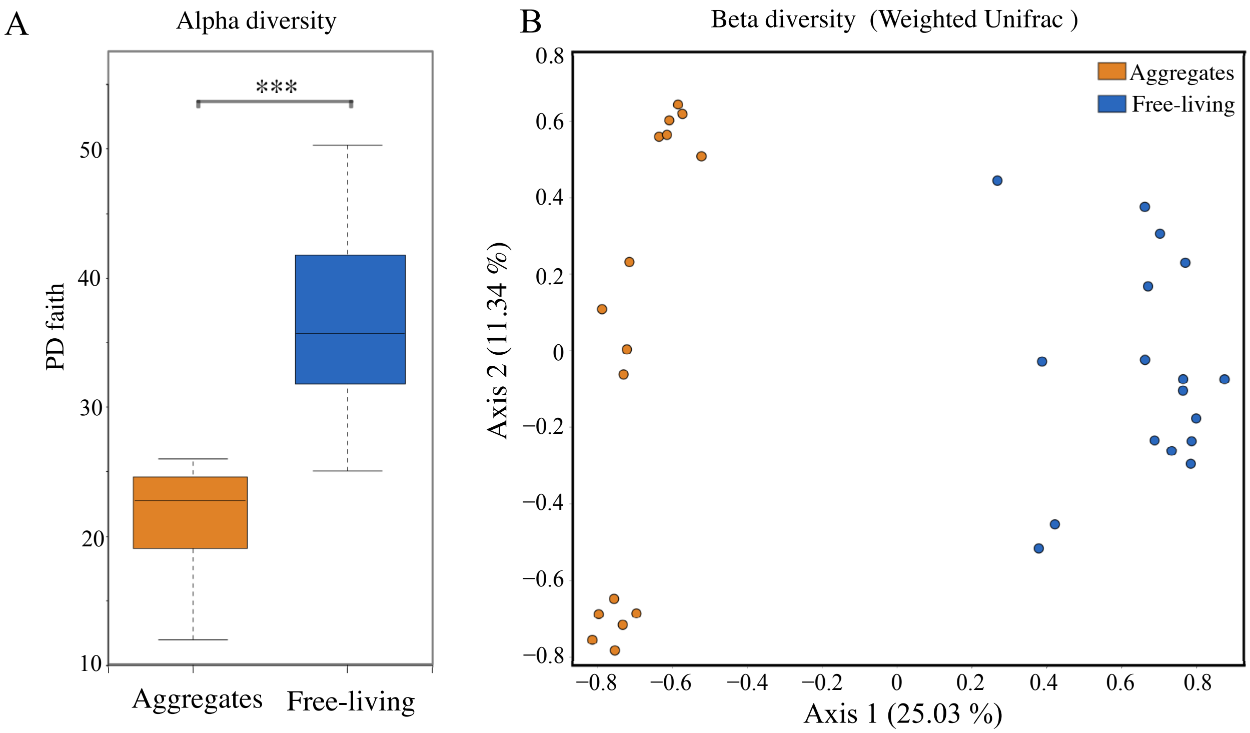
A
Alpha diversity
***
PD faith
50
40
30
20
10
Aggregates
Free-living
B
Beta diversity (Weighted Unifrac)
Aggregates
Free-living
0.8
0.6
0.4
0.2
0
−0.2
−0.4
−0.6
−0.8
Axis 2 (11.34 %)
−0.8
−0.6
−0.4
−0.2
0
0.2
0.4
0.6
0.8
Axis 1 (25.03 %)

Figure S4

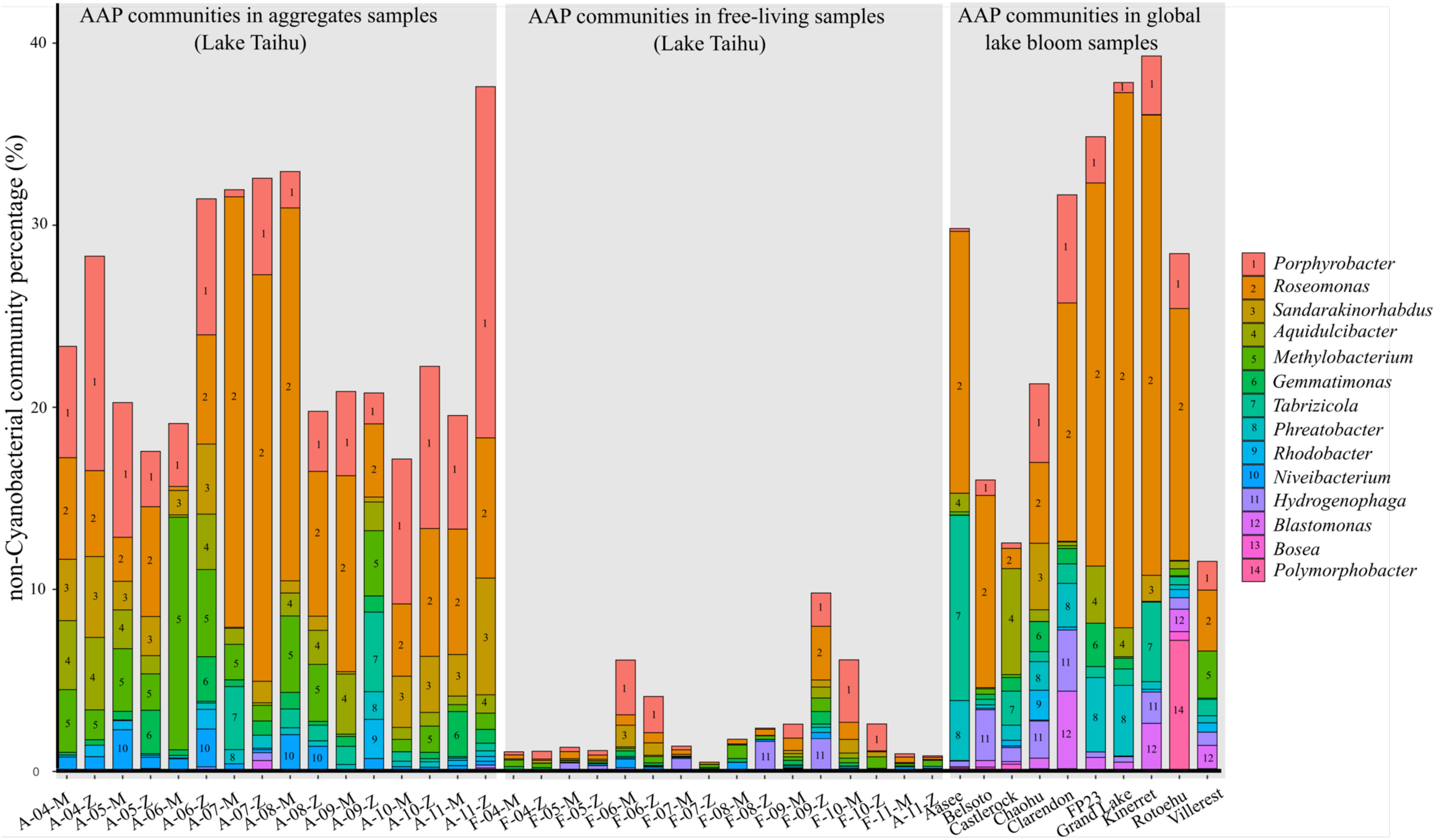
AAP communities in aggregates samples (Lake Taihu)
AAP communities in free-living samples (Lake Taihu)
AAP communities in global lake bloom samples
non-Cyanobacterial community percentage (%)
0
10
20
30
40
A-04-M
A-04-Z
A-05-M
A-05-Z
A-06-M
A-06-Z
A-07-M
A-07-Z
A-08-M
A-08-Z
A-09-M
A-09-Z
A-10-M
A-10-Z
A-11-M
A-11-Z
F-04-M
F-04-Z
F-05-M
F-05-Z
F-06-M
F-06-Z
F-07-M
F-07-Z
F-08-M
F-08-Z
F-09-M
F-09-Z
F-10-M
F-10-Z
F-11-M
A-11-Z
Aasee
Belsoto
Castlerock
Chaohu
Clarendon
FP23
Grand Lake
Kinerret
Rotoehu
Villerest
1 Porphyrobacter
2 Roseomonas
3 Sandarakinorhabdus
4 Aquidulcibacter
5 Methylobacterium
6 Gemmatimonas
7 Tabrizicola
8 Phreatobacter
9 Rhodobacter
10 Niveibacterium
11 Hydrogenophaga
12 Blastomonas
13 Bosea
14 Polymorphobacter
Figure S5

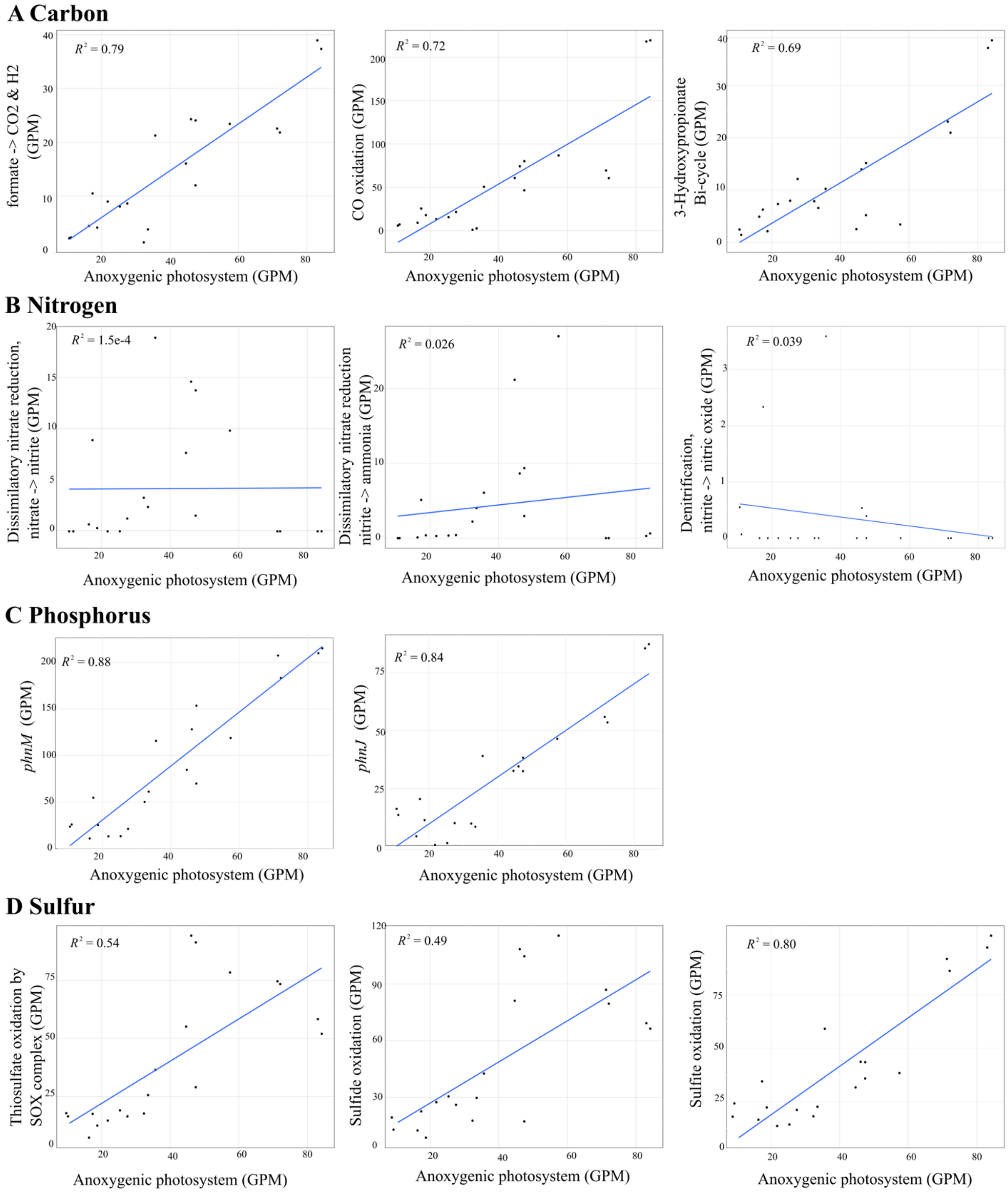
A Carbon
$R^2 = 0.79$
formate -> CO2 & H2 (GPM)
Anoxygenic photosystem (GPM)
$R^2 = 0.72$
CO oxidation (GPM)
Anoxygenic photosystem (GPM)
$R^2 = 0.69$
3-Hydroxypropionate Bi-cycle (GPM)
Anoxygenic photosystem (GPM)
B Nitrogen
$R^2 = 1.5e-4$
Dissimilatory nitrate reduction, nitrate -> nitrite (GPM)
Anoxygenic photosystem (GPM)
$R^2 = 0.026$
Dissimilatory nitrate reduction nitrite -> ammonia (GPM)
Anoxygenic photosystem (GPM)
$R^2 = 0.039$
Denitrification, nitrite -> nitric oxide (GPM)
Anoxygenic photosystem (GPM)
C Phosphorus
$R^2 = 0.88$
phnM (GPM)
Anoxygenic photosystem (GPM)
$R^2 = 0.84$
phnJ (GPM)
Anoxygenic photosystem (GPM)
D Sulfur
$R^2 = 0.54$
Thiosulfate oxidation by SOX complex (GPM)
Anoxygenic photosystem (GPM)
$R^2 = 0.49$
Sulfide oxidation (GPM)
Anoxygenic photosystem (GPM)
$R^2 = 0.80$
Sulfite oxidation (GPM)
Anoxygenic photosystem (GPM)

Figure S6
